# Highly Efficient, Spatially Pure Circularly Polarized Luminescence from Bilayer Self‐Assembled Colloidal Quantum Wells and Soft Helical Superstructures

**DOI:** 10.1002/advs.202509122

**Published:** 2025-08-13

**Authors:** Xiao Liang, Thi Thu Ha Do, Lu Ding, Furkan Isik, Anupam Sadhu, Emek G. Durmusoglu, Syed Akhil, Betul Canimkurbey, Lydia H. Wong, Son Tung Ha, Arseniy I. Kuznetsov, Hilmi Volkan Demir

**Affiliations:** ^1^ LUMINOUS! Center of Excellence for Semiconductor Lighting and Displays School of Electrical and Electronic Engineering Nanyang Technological University 50 Nanyang Avenue Singapore 639798 Singapore; ^2^ Institute of Materials Research and Engineering A*STAR (Agency for Science, Technology and Research) 2 Fusionopolis Way, #08‐03 Innovis Singapore 138634 Singapore; ^3^ School of Material Science and Engineering Nanyang Technological University 50 Nanyang Avenue Singapore 639798 Singapore; ^4^ UNAM‐Institute of Materials Science and Nanotechnology The National Nanotechnology Research Center Department of Electrical and Electronics Engineering Department of Physics Bilkent University Ankara 06800 Turkey; ^5^ Polatlı Faculty of Sciences and Arts Department of Physics Ankara Hacı Bayram Veli University Ankara 06900 Turkey

**Keywords:** angle‐resolved spectroscopy, asymmetry factor, circularly polarized luminescence, colloidal quantum wells, helical superstructure, self‐assembly

## Abstract

Circularly polarized luminescence (CPL) is important for multiple photonic technologies. It can be achieved with high asymmetry factors (*g_lum_*) by combining quantum emitters (QEs) with one‐dimensional helical superstructures (1D‐HS). However, existing 1D‐HS systems face challenges of maintaining polarization purity across viewing angles, primary due to the mismatch between QE emission profiles and the photonic bandgap of 1D‐HS across off‐normal directions. Herein, efficient and controllable CPL is proposed and developed using the self‐assembly of colloidal quantum wells (CQWs) coupled with cholesteric liquid crystals (CLCs). The face‐down CQWs assemblies with over 90% in‐plane transition dipole moments enables directional emission along the liquid crystal helical axis within the light escape cone. At the same time aligning their narrow emission spectra to the edge of the CLCs reflection band significantly enhances the spectral coupling. This results in highly efficient CPL with an improved *g_lum_* of 1.47–1.82 (±0.03) over an expanded viewing range (±40°) and a large increase (53.3%) in extraction efficiency, supported by comprehensive angle‐resolved and wavelength‐resolved spectroscopy as well as optical simulations. Moreover, this approach facilitates the development of novel anti‐peeping and angle‐dependent luminescent devices. This work establishes a versatile platform for spatially homogeneous and tunable CPL in next‐generation photonic systems.

## Graphical abstract

Highly efficient and spatially pure circularly polarized luminescence (CPL) is achieved by coupling face‐down self‐assembled colloidal quantum wells with cholesteric liquid crystals. This design yields a high *g_lum_* (up to 1.82) over a wide viewing angle (±40°), supported by comprehensive angle‐ and wavelength‐resolved spectroscopy and optical simulations. This approach provides a versatile platform for advancing CPL‐based photonic technologies.

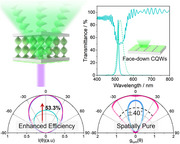

## 1 Introduction

Circularly polarized luminescence (CPL), distinguished by its ability to generate and encode distinct polarization states of emitted light, plays a pivotal role in emerging photonic technologies,^[^ ^1^ ^]^ encompassing secure optical encryption,^[^ ^2^ ^]^ anti‐counterfeiting,^[^ ^3^ ^]^ optoelectronics,^[^ ^4^ ^]^ 3D display systems,^[^ ^5^ ^]^ and more. A critical metric for assessing the efficacy of CPL is the asymmetry factor (*g_lum_*), defined as *g_lum_* = 2(*I_L_* − *I_R_*)/(*I_L_* + *I_R_*), where *I_L_* and *I_R_* denote the intensities of left‐ and right‐handed circularly polarized light, respectively.^[^ ^6^ ^]^ Among the diverse approaches for achieving CPL, inorganic quantum emitters (QEs) such as II‐VI quantum dots (QDs) and perovskite QDs, have attracted significant attention, thanks to their superior chemical and photophysical stability compared to the organic counterparts, as well as their tunable luminescence properties.^[^ ^1^, ^7^ ^]^ To date, various material strategies and design paradigms have been explored, such as surface functionalization of QEs with chirality moieties,^[^ ^8^ ^]^ self‐assembly of QEs into higher‐order chiral architectures,^[^ ^9^ ^]^ and the integration of QEs with chiral systems.^[^ ^7^, ^10^ ^]^ Notably, the incorporation of QEs with one‐dimensional helical superstructures (1D‐HS) that feature a periodically varying dielectric constant along the optical axis—such as cholesteric liquid crystals (CLCs).^[^ ^7^, ^11^ ^]^ and cellulose nanocrystals^[^ ^12^ ^]^ has demonstrated exceptional CPL performance, achieving the highest reported *g_lum_*, reaching up to 1.9 at normal incidence.^[^ ^7c^ ^]^

Despite the promising performance of QEs/1D‐HS hybrid systems, several critical challenges persist. Chief among these is the pronounced angular dependence of the optical bandgap in 1D‐HS structures.^[^ ^7b^ ^]^ Current design strategies typically align the emission peak of the QEs with the central wavelength of the 1D‐HS photonic bandgap,^[^ ^13^ ^]^ ensuring high‐quality CPL at normal incidence. However, the inherent angular shift of the photonic bandgap causes a spectral mismatch at oblique angles, diminishing the asymmetry factor. This issue is further exacerbated by the broader spectral emission profiles of QDs, as the degree of overlap between the QDs emission spectrum and the photonic bandgap rapidly decreases with increasing viewing angle. For longer‐wavelength emitting QDs, such as red‐emitting ones, the relatively larger cores undergo a size‐focusing stage during growth,^[^ ^14^ ^]^ partially mitigating size distribution disparities. Conversely, shorter‐wavelength QDs, such as green‐emitting ones, are much smaller relative to their Bohr exciton radius, resulting in broader size distributions and consequently wider emission linewidths. Furthermore, the alternating high‐ and low‐refractive‐index layers in the 1D‐HS structure inherently reduce light extraction efficiency due to increased total internal reflection and optical losses. These factors collectively result in low CPL efficiency and a rapid degradation of polarization purity at oblique angles, limiting the practical applicability of current QEs/1D‐HS hybrid systems.

Realizing efficient and spatially homogeneous CPL with enhanced asymmetry factors is crucial for numerous advanced applications that demand spectral and angular versatility. For instance, in optical encryption systems, narrow spectral bandwidth limits data density and reduces system resilience.^[^ ^15^ ^]^ Similarly, in 3D display technologies, low degree of polarization introduces undesirable color distortion and narrows the effective viewing zone, compromising user experience.^[^ ^16^ ^]^ Achieving robust CPL with spatially high *g_lum_* across broad spectral bandwidth remains an unresolved challenge, stemming from the limited 3D interaction between the QEs emission profile and the photonic bandgap of the chiral photonic crystals. To overcome these challenges, a systematic and multifaceted approach is essential. First of all, the development of advanced characterization methodologies is essential, as precise evaluation of asymmetry factors across the full spectral and angular ranges provides critical insights into CPL performance and guides subsequent optimization efforts. Moreover, since the light escape cone serves as the sole interface connecting light propagation between the internal medium and free space, achieving highly efficient and spatially pure CPL depends on maximizing the proportion of light falling within the escape cone while simultaneously ensuring effective CPL conversion between QEs and CLCs within this spatial region. This requires engineering QEs to exhibit directional emission profiles along the optical axis within the escape cone and narrowing their emission linewidths to enhance coupling with the photonic bandgap of the CLCs across varying angles.

Based on the strategy mentioned above, herein we present a novel bilayer configuration consisting of all face‐down self‐assembled colloidal quantum wells (CQWs) stacked with CLCs to tackle these challenges, and the CPL performance is comprehensively characterized in both spectral and spatial domains by using a home‐built angle‐resolved and wavelength‐resolved spectroscopy system.^[^ ^17^ ^]^ Quasi‐2D CQWs were employed as the QEs thanks to their atomically smooth surfaces and highly uniform quantum confinement, which eliminate spectral inhomogeneities associated with size distributions.^[^ ^18^ ^]^ Advanced heterostructure engineering enabled the synthesis of high‐efficiency CQWs with narrow emission linewidths spanning the visible spectrum, providing a versatile 2D QEs material platform for spatially pure CPL with full spectral coverage. Through self‐assembly techniques, we achieved large‐area alignment of CQWs in an all face‐down configuration, ensuring in‐plane transition dipole moments (TDMs) orientation and enhanced directional emission. Optical simulation based on the finite‐difference time‐domain (FDTD) method revealed that the in‐plane orientation of TDMs in the CQWs/CLCs stacking led to a theoretical 53.3% increase in extraction efficiency compared to QDs/CLCs counterparts, and the ultra‐narrow emission photoluminescence (PL) aligned near the left‐hand edge of the CLCs reflection band mitigated angle‐dependent blue shifts, greatly improving spectral and angular coupling within the light escape cone. Consequently, we achieved efficient and wide‐angle CPL with an improved peak *g_lum_* of 1.47–1.82 (±0.03) over an expanded viewing angle range exceeding ±40°, significantly outperforming conventional QDs/1D‐HS systems with a peak *g_lum_* of ≈1.5, and which drop rapidly to below 0.4 at ±40°. Finally, we expand the versatility of our design to develop a novel 360° omnidirectional anti‐peeping device and a device with angular‐dependent emission properties, demonstrating potential applications in the creation of intelligent optical systems. These results lay the groundwork in terms of design strategies, characterization techniques, and material platforms, for further advancements in CPL‐based photonic technologies

## 2 Results and Discussion

**Figure 1:**
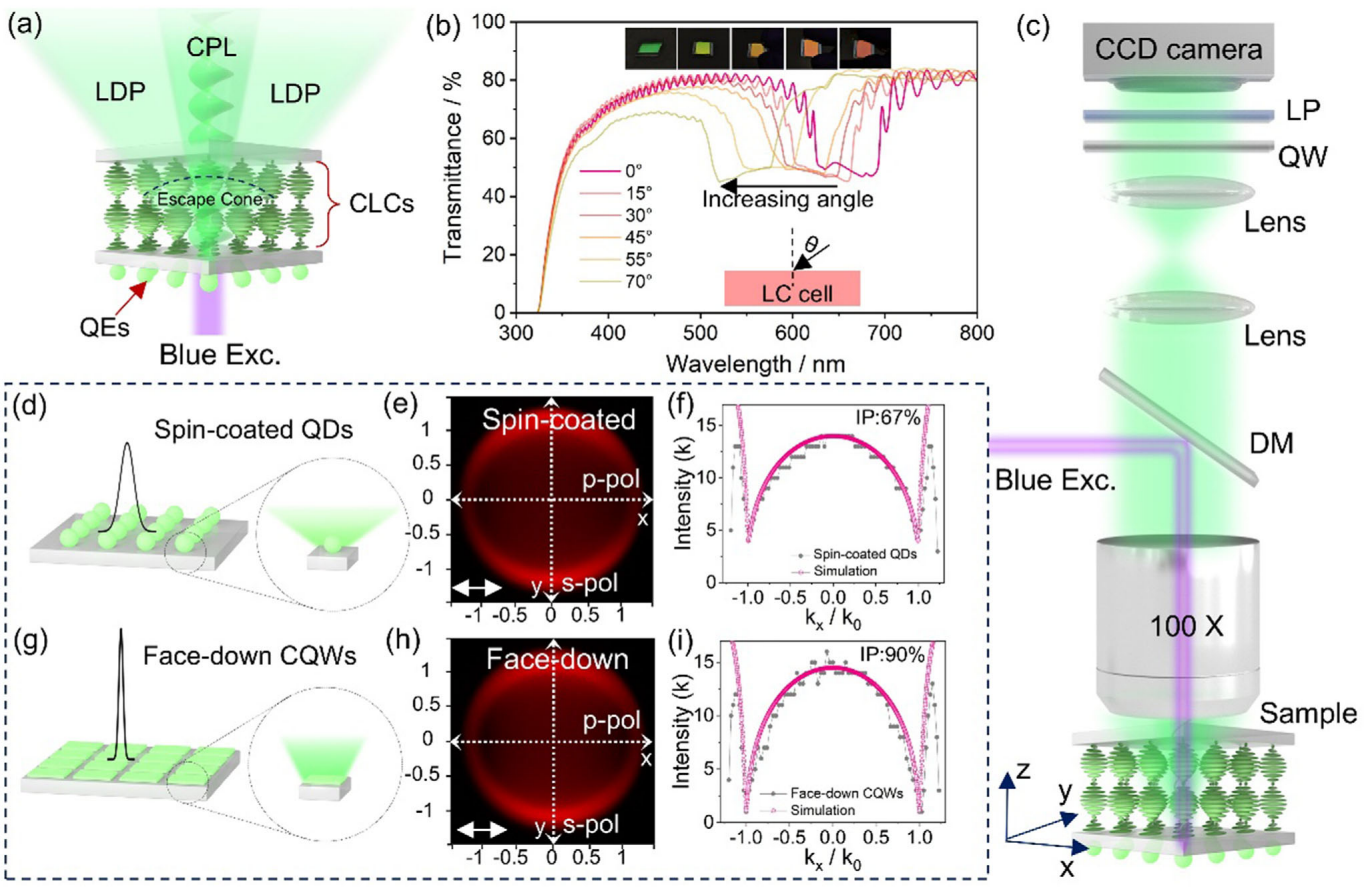
Strategies for achieving pure and efficient CPL in a hybrid QE/CLCs configuration. a) Schematic illustration of the bilayer structure composed of QEs and planar‐oriented CLCs, showing a low degree of polarization (LDP) in the emitted light at oblique angles. b) Angle‐dependent reflection of a planar‐oriented CLCs sample. Inset: Photographs of the CLC films taken at different viewing angles. c) Schematic illustration of the angle‐resolved and wavelength‐resolved spectroscopy system. Schematic illustrations for d) spin‐coated QDs with a conventional Lambertion emission and g) face‐down self‐assembled CQWs with a directional emission. BFP images of (e) spin‐coated QDs and h) face‐down self‐assembled CQWs, respectively. Cross‐sectional intensity profiles along *k_x_*/*k*_0_ (black dotted lines) for the f) spin‐coated QDs and i) face‐down self‐assembled CQWs, respectively, fitted (red curves) following the model in Ref [20].

**Figure** **1**a illustrates the general stacking architecture of a bilayer system comprising QEs and planar CLCs. To achieve CPL with a high *g_lum_*, it is critical to align the emission spectra of the QEs within the selective Bragg reflection band of the CLCs.^[^ ^13^ ^]^ The self‐organized helical superstructure of the CLCs with one single‐handedness acts as an efficient circularly polarized light filter, selectively transmitting circularly polarized light with the opposite handedness. However, it is important to note that the reported high *g_lum_* values for QDs in literature are typically calculated around normal incidence and for a specific wavelength (e.g., the emission peak) as per conventional characterization methods outlined in prior studies (Figure S1, Supporting Information). For most 1D chiral photonic crystals, the selective reflection band exhibits a pronounced angular dependence, as shown in Figure 1b and Figure S2 (Supporting Information). This behavior arises from the reduction in the effective pitch as light propagates through the helical structure of the CLCs at larger incident angles, resulting in a blue shift of the selective reflection band with increasing angle of incidence.^[^ ^19^ ^]^ As a result, emission spectra and reflection bands that are originally designed to match at normal incidence become increasingly misaligned at oblique angles, especially within the light escape cone highlighted in Figure 1a, leading to a decline in the CPL asymmetry factor in the free space. Furthermore, traditional methods often fail to account for the spectral emission bandwidth of QEs. Factors such as the dispersion broadening caused by the size distribution of QDs and their Lambertian radiation patterns further exacerbate mismatches with the angle‐dependent bandgap of 1D‐HS, accelerating the decrease in the asymmetry factor. Therefore, the first priority is to establish an effective method to comprehensively collect the full spectral and angular distribution of the emitted light and its polarization states from the QEs.

To capture the polarization information of emitted light across different angles and wavelengths, we built an optical setup based on angle‐resolved and wavelength‐resolved spectroscopy, as illustrated in Figure 1c. The blue excitation filtered from a mercury lamp is guided to a dichroic mirror (DM) and focused on the sample through an objective with a high numerical aperture (NA = 0.7). The emitted light was collected by the same objective, and passed through a lens system that resolves the angular distribution of the emission at the spectrometer. In order to calculate the asymmetry factor *g_lum_*_,_ we measured the angle‐resolved PL with left‐handed and right‐handed polarization by using a pair of a quarter waveplate (QW) and a linear polarizer (LP, analyzer) set up along the detection path, and computed *g_lum_* = 2(*I_L_* − *I_R_*)/(*I_L_* + *I_R_*)_._ For full access to all the angles in the *x*, *y*‐plane (θ_*x*_,θ_*y*_), the entrance slit to the spectrometer was opened fully, whereas it was closed to 100 µm for angle‐resolved PL spectra. This technique allows for a comprehensive evaluation of the spectral and angular dependence of *g_lum_*. Unlike conventional methods, which often overlook angular variations or are limited to specific wavelengths, our approach can provide deeper insights into the CPL performance of QEs integrated with photonic structures.

To achieve highly efficient and spatially pure CPL emission, it is imperative to increase the proportion of light emitted from QEs that is directed toward the escape cone, while simultaneously enabling CPL conversion of unpolarized light within this region through effective interaction with CLCs. Conventional spherical emitters, such as QDs, typically exhibit Lambertian radiation patterns due to their randomly oriented TDMs (Figure 1d).^[^ ^21^ ^]^ In this study, we used the back focal plane (BFP) imaging technique with an oil‐immersion objective (NA = 1.2) for evaluating the spatial radiation properties of the QEs samples by resolving the orientation distribution of transition dipoles, as shown in Figure S5 (Supporting Information), where each point in the BFP pattern corresponds to a unique emission angle determined by the photon momentum *k* and the linear polarizer. The BFP patterns and the cuts along *k_x_* for the spin‐coated QDs are presented in Figure 1e,f. Here we focus on *k_x_*, as the relative contributions of in‐plane (IP) and out‐of‐plane (OP) dipoles—indicative of dipole anisotropy—are revealed exclusively in p‐polarized emission. It was previously reported that a distinctive feature of emission from 100% in‐plane dipoles is the zero‐intensity observed at *k_x_* = ±*k*_0_, arising from complete destructive interference of the field components.^[^ ^20^ ^]^ As the OP contribution increases, the intensity at these angles correspondingly rises, as further confirmed by our simulation results (Figure S6, Supporting Information). For the spin‐coated QDs, the IP dipole proportion is calculated to be 67% (Figure 1f), consistent with theoretical predictions for two degenerate IP dipole orientations (x and y directions) and one OP dipole orientation (z direction). In contrast, by using quasi‐2D CQWs, which possess anisotropic TDMs, directional emission can be realized by achieving in‐plane orientation of TDMs through all face‐down self‐assembly (Figure 1g),^[^ ^22^ ^]^ in which all CQWs adopt a flat orientation with their broad surfaces facing the substrate (Figure S7a, Supporting Information). This approach has recently been demonstrated as an effective strategy to significantly enhance the external quantum efficiency of light‐emitting diodes.^[^ ^23^ ^]^ The BFP image and the analysis of the self‐assembled CQWs indicates an IP dipole fraction exceeding 90% (Figure 1h,i), aligning with previous experimental results in lasing and LED research.^[^ ^23^, ^24^ ^]^ These observations confirm that an all‐face‐down oriented CQWs film significantly increases the proportion of emitted light that falls within the light escape cone. To complete the process of achieving spatially pure CPL emission, it is essential to efficiently convert the unpolarized light within the escape cone into CPL. This is facilitated by another advantageous characteristic of CQWs—their ultranarrow emission linewidth (Figure 1g)—which enables effective coupling with the optical bandgap of the CLCs. This spatial coupling allows for the highly efficient transformation of unpolarized light within the escape cone into CPL, which is subsequently emitted into free space. This synergistic design strategy holds great promise for achieving highly efficient and spatially pure CPL emission.

**Figure 2:**
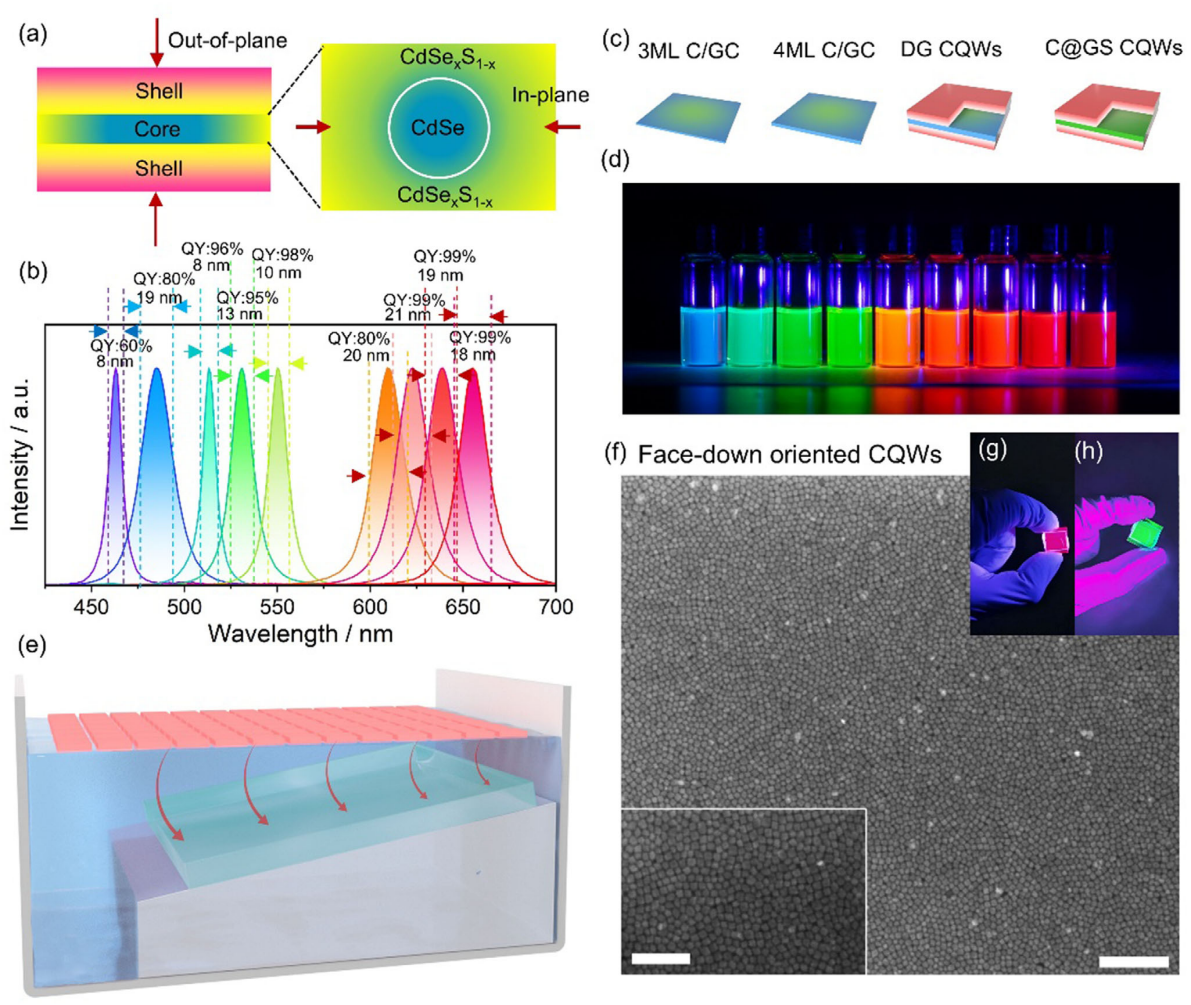
Development of spectrally tunable CQWs with high quantum yield and face‐down self‐assembly technique. a) Schematics of the gradient heterostructure CQWs:the cross‐sectional view of the core@shell structure (left) and the planar structure of the core‐gradient crown (right). b) PL spectra of the as‐synthesized CQWs, with the corresponding FWHM and QY indicated in the figure. c) Representative schematics·of the CQWs emitting different PL spectra: deep blue (3ML C/GC), green (4ML C/GC), red (DG CQWs) and deep red (C@GS CQWs). d) Photograph showcasing the full PL spectral coverage, spanning from deep blue to deep red. e) Schematic illustration of the liquid‐liquid interface self‐assembly process used to fabricate orientation‐controlled self‐assembled monolayer CQWs. f) Scanning electron microscopy (SEM) image of a large‐area face‐down self‐assembled CQWs film (scale bar: 250 nm). Inset: magnified SEM image with a scale bar of 100 nm. g,h) Photographs of the as‐prepared self‐assembled CQWs films under UV (405 nm) light excitation.

Based on the above design strategy, the development of spectrally tunable quasi‐2D CQWs with a high QY represents a crucial step toward achieving efficient CPL with high spatial purity. In this work, we present a series of highly efficient CQWs with full‐visible spectra coverage via incorporating gradient heterostructures along lateral and/or vertical directions of CdSe cores with varying atomic‐layer thicknesses (3, 4, and 5 monolayers), as shown in **Figure** **2**a. These CQWs exhibited high QYs (as high as 99%) and ultra‐narrow emission linewidths (from 8‐18 nm) across the red (653 nm), green (514‐549 nm), and blue (463 nm) spectral regions, as summarized in Figure 2b (Movie S1, Supporting Information), with their structural schematics and samples image shown in Figure 2c–d. Absorption and PL spectra of the corresponding CQWs are revealed in Figures S8–S10 (Supporting Information), and transmission electron microscopy (TEM) characterization results are provided in Figure S11 (Supporting Information). For the blue (463 nm) and green (514–549 nm) CQWs, a core‐gradient crown structure was employed, consisting of a CdSe core with a compositionally graded CdSe_x_S_1‐x_ crown that extends laterally, using protocols reported in the literature.^[^ ^25^ ^]^ This gradient crown effectively passivates the nonpolar edge facets, where ligands are weakly bound and prone to detachment, which can introduce trap states and reduce the QY.^[^ ^26^ ^]^ Additionally, the surface of the CQWs is terminated by polar (100) facets composed of a monolayer of cationic sites (Cd^2^⁺), which are tightly bound to long‐chain carboxylate ligands, effectively passivating surface trap states.^[^ ^27^ ^]^ This excellent passivation on both the top/bottom surfaces and the side facets results in exceptionally high QYs up to 98%, as further supported by the near mono‐exponential decay profiles observed in fluorescence lifetime measurements (Figure S12, Supporting Information). Moreover, the uniform 1D quantum confinement ensures the ultra‐narrow full‐width at half‐maximum (FWHM) of 8–13 nm in these heterostructured CQWs. For red emission at 653 nm, a gradient shell composed of Cd_x_Zn_1‐x_S was grown on the core‐gradient crown structure, achieving a FWHM of 18 nm and a near‐unity QY. Adjusting the CdSe core size or modifying the shell composition enabled emission at additional wavelengths but at the cost of introducing some spectral broadening. For instance, employing a pure ZnS shell shifted the emission peak to 600–620 nm, accompanied with an increased FWHM of 21 nm, attributed to lattice mismatch between CdSe and ZnS, which enhances phonon scattering induced spectral diffusion. Overall, the PL properties of this 2D QE system, particularly its narrow linewidth and intrinsic anisotropy TDMs, largely outperform conventional QD systems, making it an ideal candidate for high‐performance CPL applications.

To achieve face‐down oriented self‐assembly of 2D CQWs, various techniques, such as inkjet printing and evaporation deposition, have been employed. Here, we utilized a liquid‐air interface self‐assembly method,^[^ ^28^ ^]^ enabling the deposition of a self‐assembled layer of CQWs in an all‐face‐down configuration over a large, uniform area across centimeters. In this process, as shown in Figure 2e, 20 µL of a hexane solution containing CQWs was carefully deposited onto an acetonitrile (ACN) subphase. Upon hexane evaporation, the CQWs formed a uniform all‐face‐down film, which was subsequently transferred onto planar‐oriented CLCs substrates by controlled ACN drainage through a needle at the container's base (Figure S13, Supporting Information). Notably, the solvent evaporation rate critically influences the uniformity and configuration, particularly for thinner CQWs with a rectangular shape, which tend to form aggregates or stackings as revealed by previous results.^[^ ^29^ ^]^ Accelerating evaporation by increasing ambient temperature effectively prevents the CQWs from aggregating or transitioning to the thermodynamically favorable edge‐up configuration, as they are rapidly trapped by the solvent front during their dispersion across the ACN surface.^[^ ^30^ ^]^ Figure 2f and Figure S14 (Supporting Information) show SEM and TEM images of self‐assembled CQWs with varying thicknesses and shapes, demonstrating the consistent formation of large‐area, uniform all‐face‐down self‐assembled films.

**Figure 3:**
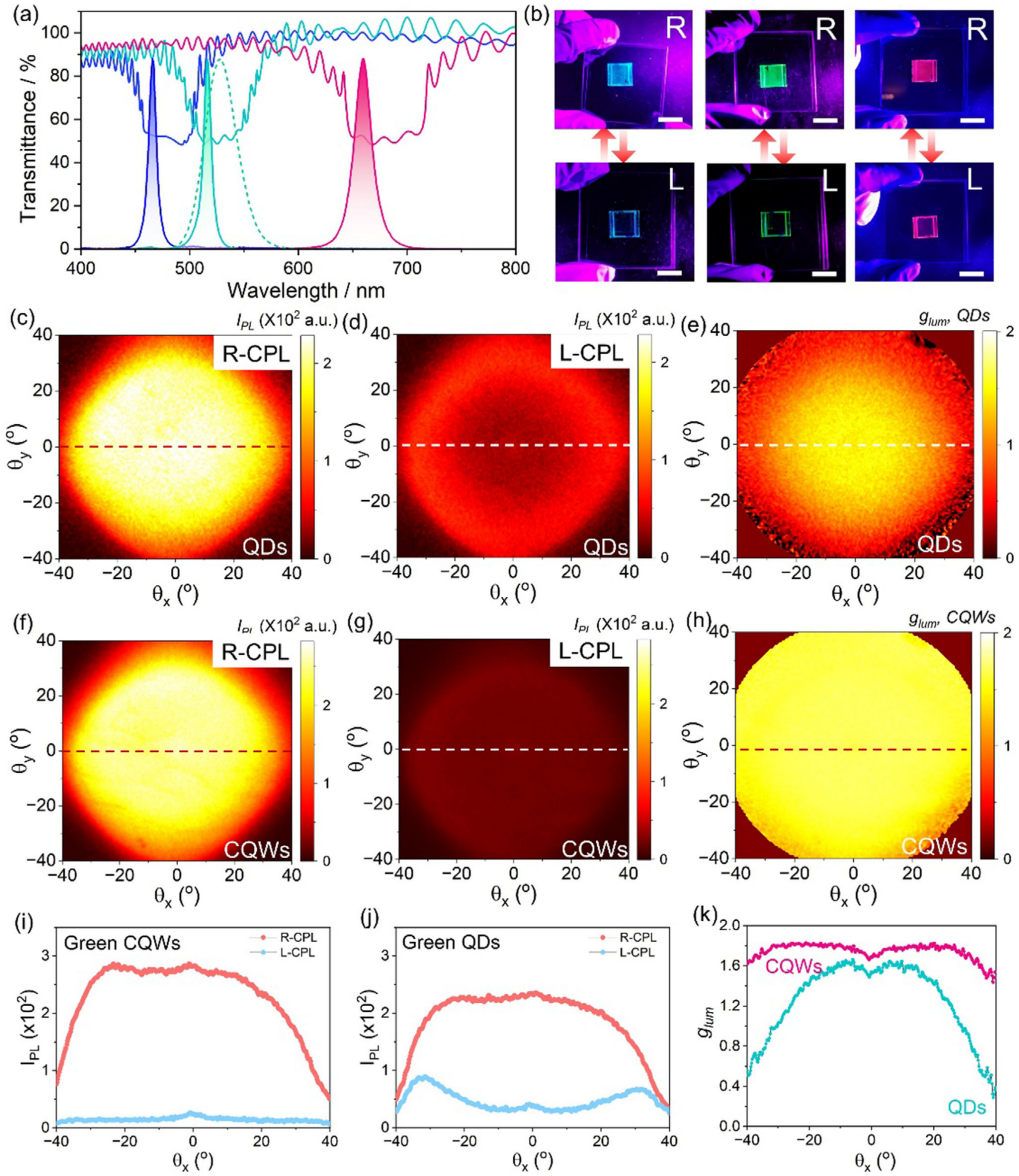
Bilayer of CQWs/CLCs for CPL with high spatial purity. a) Alignment of the LCs reflection band with the PL spectra of CQWs emitting blue, green, and red CPL. The green dotted line represents the PL spectrum of a green QDs sample for comparative study. b) Photographs of CPL from the as‐fabricated samples, taken through right‐handed (top) and left‐handed (bottom) circular polarizers (Scale bar: 1 cm). BFP images of the QDs/CLCs sample, filtered through right‐handed c) and left‐handed d) circular polarizers, respectively. e) Calculated *g_lum_* for the QDs/CLCs sample. BFP images of the CQWs/CLCs sample, filtered through right‐handed f) and left‐handed g) circular polarizers, respectively. h) Calculated *g_lum_* for the CQWs/CLCs sample. Cross‐sectional intensity profiles along θ_x_ (dash lines in BFP images) for the i) CQWs/CLCs and j) QDs/CLCs samples. k) Comparison of θ_*x*_–dependent *g_lum_* variations between the CQWs/CLCs and QDs/CLCs samples.

To further optimize the spatial overlap between the emission spectra of CQWs and the angle‐dependent photonic bandgaps of CLCs, we precisely tuned the reflection spectrum of the LCs by adjusting the concentration of the chiral dopant. Given that the reflection spectrum of CLCs exhibits a blue shift as the observation angle increases, aligning the short‐wavelength edge of the reflection band with the PL spectra of CQWs allows for enhanced spectral coverage across various angles, as shown in **Figure** **3**a. Based on this design principle, we fabricated self‐assembled CQWs/CLCs samples capable of emitting CPL at blue (463 nm), green (514 nm), and red (653 nm) wavelengths, as illustrated in Figure 3a. Since the chiral dopant S1011 used in the CLCs induces left‐handed helical superstructures, all the samples emit right‐handed circularly polarized light. When paired with a left‐handed circular polarizer, effective extinction of the emitted light can be achieved, as shown in Figure 3b. Furthermore, using the angle‐resolved and wavelength resolved spectroscopy, we investigated the angular tolerance of the samples. For comparative analysis, core@shell (CdSe_x_S_1‐x_@ZnS) QDs, with a FWHM of 33 nm and a QY of 80% (Figure S15, Supporting Information), were synthesized following established protocols in the literature.^[^ ^31^ ^]^ The central reflection wavelength of the CLCs in the QDs/CLCs samples coincided with the PL peaks (535 nm) of the QDs (dotted lines, Figure 3a) to better match the QDs broader emission profiles. Figure S16 (Supporting Information) presents the results of a control sample consisting of a spin‐coated layer of QDs on a quartz substrate, revealing a circular dichroism of less than 2.5% (*g_lum_* < 0.05), which is attributed to the response of our setup, as the emission of the QDs is completely unpolarized.^[^ ^32^ ^]^ Figure 3c,d present the angular distribution of left‐ and right‐handed CPL intensities of the QDs samples with the incorporation of CLCs, with the corresponding *g_lum_* calculated in Figure 3e. Accounting for the full emission spectrum and angular effects, the *g_lum_* at normal incidence was moderate at ≈1.5, limited by the broad emission profile of the QDs, where longer‐wavelength components fall outside the photonic bandgap of the CLCs, as indicated by the wavelength‐resolved characterizations in Figure S17d (Supporting Information). Additionally, *g_lum_* dropped sharply to below 0.4 at oblique angles of ±40° due to the mismatch between the Lambertian emission of QDs and the angular‐dependent photonic bandgap of the CLCs (Figure 3k). These results indicate that only a small fraction of light emitted by the QDs, primarily concentrated in the normal direction, is partially converted into CPL. This limitation in efficiency and angular dependence poses challenges for practical applications that demand higher spatial purity. In contrast, Figure 3f,g presents the angular distribution of left‐ and right‐handed CPL intensities of CQWs/CLCs samples, with the corresponding *g_lum_* calculated in Figure 3h. The CQWs/CLCs sample demonstrates an enhanced peak *g_lum_* of ≈1.65, while maintaining a *g_lum_* range of 1.47 to 1.82 (±0.03) over a broad viewing angle exceeding ±40°. Similar results were also obtained in CQWs emitting at other wavelengths, e.g., 550 nm and 610 nm, as shown in the Supporting Information (Figures S18, S19, Supporting Information). Leveraging the ultranarrow emission linewidth and directional emission of CQWs, the combination of face‐down CQWs and planar CLCs enables efficient conversion of the emitted light from CQWs into CPL across different angles.

**Figure 4:**
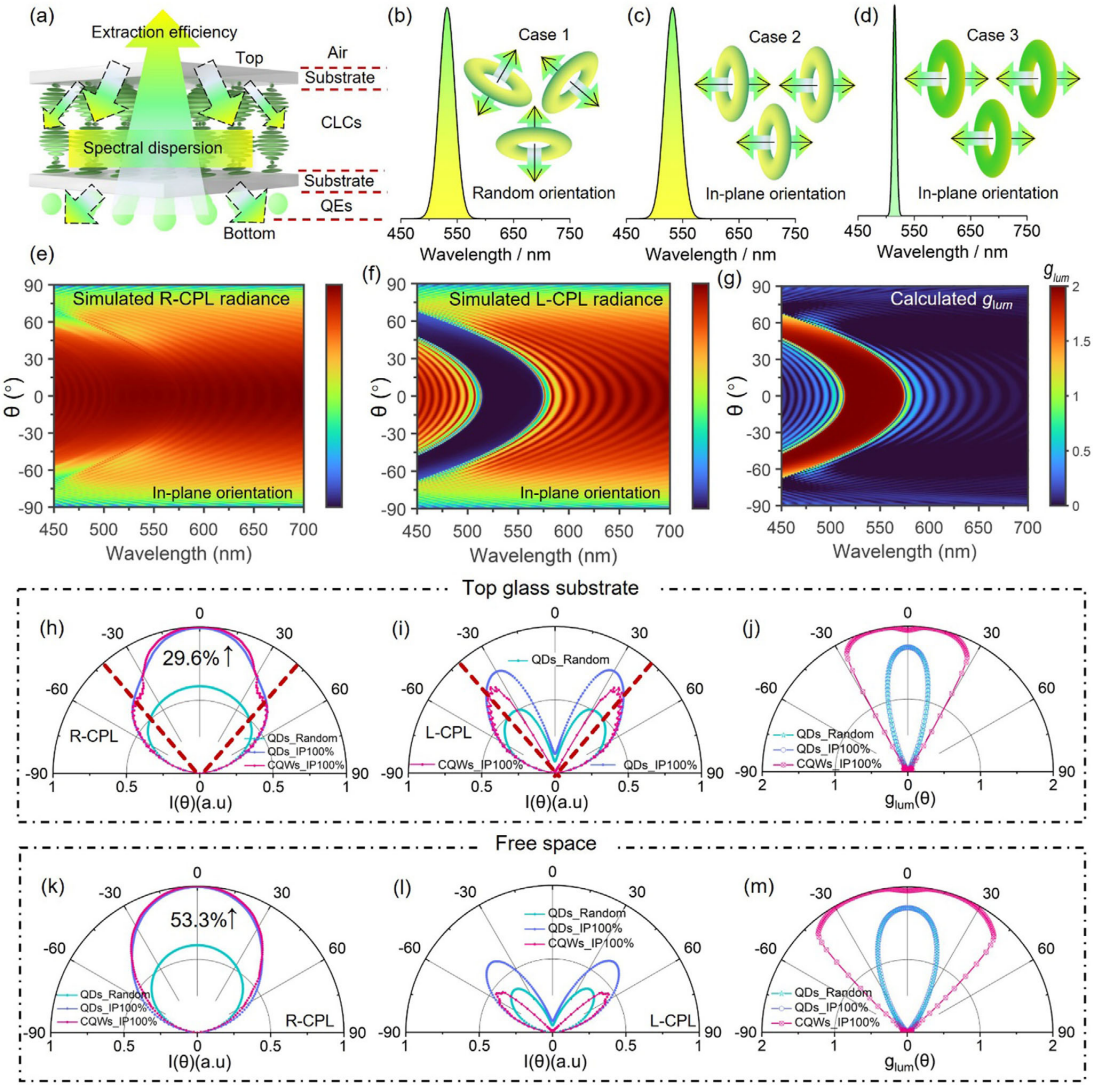
Optical simulations. a) Schematic illustrating the issues related to spectral dispersion and extraction efficiency of CPL analyzed in our simulation studies. Three scenarios of QEs were considered for comparative purposes: b) Conventional QDs with broad emission and randomly oriented TDMs (Case 1). c) An intermediate state where the QEs exhibit a broad emission linewidth similar to conventional QDs but with 100% in‐plane TDM orientation (Case 2), d) CQWs with ultra‐narrow emission and 100% in‐plane TDM orientation (Case 3). Simulated radiance of e) R‐CPL and f) L‐CPL from QEs/CLCs with 100% in‐plane TDMs in the visible spectrum. g) Calculated *g_lum_* derived from the R‐CPL and L‐CPL results. h–j) Simulated radiation patterns for (h) R‐CPL, i) L‐CPL with the red dashed lines indicating the light escape cone within ±43°, and j) *g_lum_*, incorporating QEs from the three aforementioned cases within the medium of top glass substrate. k–m) Simulated radiation patterns for k) R‐CPL, l) L‐CPL, and m) *g_lum_*, incorporating QEs from the three cases above within the medium of top glass substrate.

To gain deeper insights into the impact of dipole orientation and emission linewidth on the CPL characteristics (**Figure** **4**a), we performed optical simulations using the finite‐difference time‐domain (FDTD) method. Three distinct scenarios were devised to enable a controlled, single‐variable comparison on the individual contributions of TDM orientation and emission linewidth, as schematically illustrated in Figure 4b–d. Case 1 and Case 3 correspond to the QDs/CLCs and CQWs/CLCs from our earlier experimental conditions, respectively. To bridge these two extremes, we artificially introduced an intermediate state, Case 2, where the QEs exhibit a broad emission linewidth similar to QDs but possess a 100% IP TDM orientation akin to face‐down self‐assembled CQWs. These computational experiments enabled a rigorous decoupling of the effects of emission linewidth and TDM orientation on CPL performance. Figure 4e,f shows the representative calculated radiance for left‐handed CPL (L‐CPL) and right‐handed CPL (R‐CPL) within the medium of top glass substrate across the visible spectrum under 100% IP dipole orientation. Radiance patterns in free space or under other dipole orientations are collectively shown in Figures S22, S23 (Supporting Information). (Detailed calculation methods for optical simulation are provided in the Supporting Information). The calculated *g_lum_*, derived from the simulated L‐CPL and R‐CPL results, is presented in Figure 4g. To ensure an unbiased analysis, the simulations normalize the source power, ensuring that the 2D radiance mappings contain only the information about the optical band structure from CLCs and the dipole orientation from QEs, independent of absolute emission intensity or spectral shape. This allows for a comparison of radiation intensities under equivalent total emission power for QEs with different spectral profiles by multiplying the radiance with the corresponding emission spectrum. Figure 4h–j display the angular‐dependent simulation results for R‐CPL, L‐CPL, and *g_lum_* within top glass substrate for the three QE cases. Figure 4h illustrates that the IP alignment of TDM enhances the radiation intensity of the targeted handedness (R‐CPL in this case) by 29.6% after the PL propagates through the bottom substrate and CLCs layers, with a larger proportion of R‐CPL entering the light escape cone within ±43° (red dash lines) as compared to the QDs/CLCs. On the other hand, Figure 4i shows that the ultranarrow emission linewidth of the CQWs enhances the spatial coupling of the opposite handedness (L‐CPL) with the CLCs, evidenced by a delayed increase in L‐CPL intensity around the edges of the escape cone. The higher spatial intensity contrast between R‐CPL and L‐CPL within the light escape cone results in a higher *g_lum_* with expanded view angles, as shown in Figure 4h. These distinct yet synergistic effects of TDM orientation and narrow emission linewidth together enable the efficient and spatially pure CPL in free space, as the corresponding results calculated using Fresnel equations are summarized in Figure 4k–m. The final total efficiency enhancement in free space is determined to be 53.3% (Figure 4k), with only a minimal portion of L‐CPL escaping at very large viewing angles, leading to a much wider emission angle for high‐purity CPL, as further highlighted in Figure 4m. This simulation study conclusively demonstrates that 100% in‐plane TDM orientation maximizes the proportion of light within the escape cone, while the ultranarrow emission linewidth of CQWs enhances spectral coupling with the CLCs, ensuring effective CPL conversion within this region. To further experimentally demonstrate the synergistic effects of controlled in‐plane dipole orientation—which determines the intensity of light entering the escape cone, and optimized spectral coupling—which governs the conversion efficiency of CPL (purity) within the escape cone, we additionally prepared all edge‐up self‐assembled CQWs/CLCs. TEM characterization confirming the edge‐up orientation of the CQWs is shown in Figure S24a,b (Supporting Information). Angular CPL measurements (Figure S24c–f, Supporting Information) reveal that both samples exhibit comparable spatial CPL purity (Figure S24e, Supporting Information), owing to identical spectral overlap between the emission spectra of the same CQWs and the photonic bandgap of the CLCs. However, the R‐CPL intensity differs significantly between the two self‐assembly cases, with the edge‐up configuration showing a markedly lower intensity (Figure S24f, Supporting Information). This is attributed to the reduced fraction of in‐plane dipoles in the edge‐up assembly, resulting in weaker emission directed into the escape cone. This work represents the first demonstration of imparting highly efficient and high‐purity CPL characteristics to CdSe‐based colloidal semiconductor nanoplatelets using CLCs.

**Figure 5:**
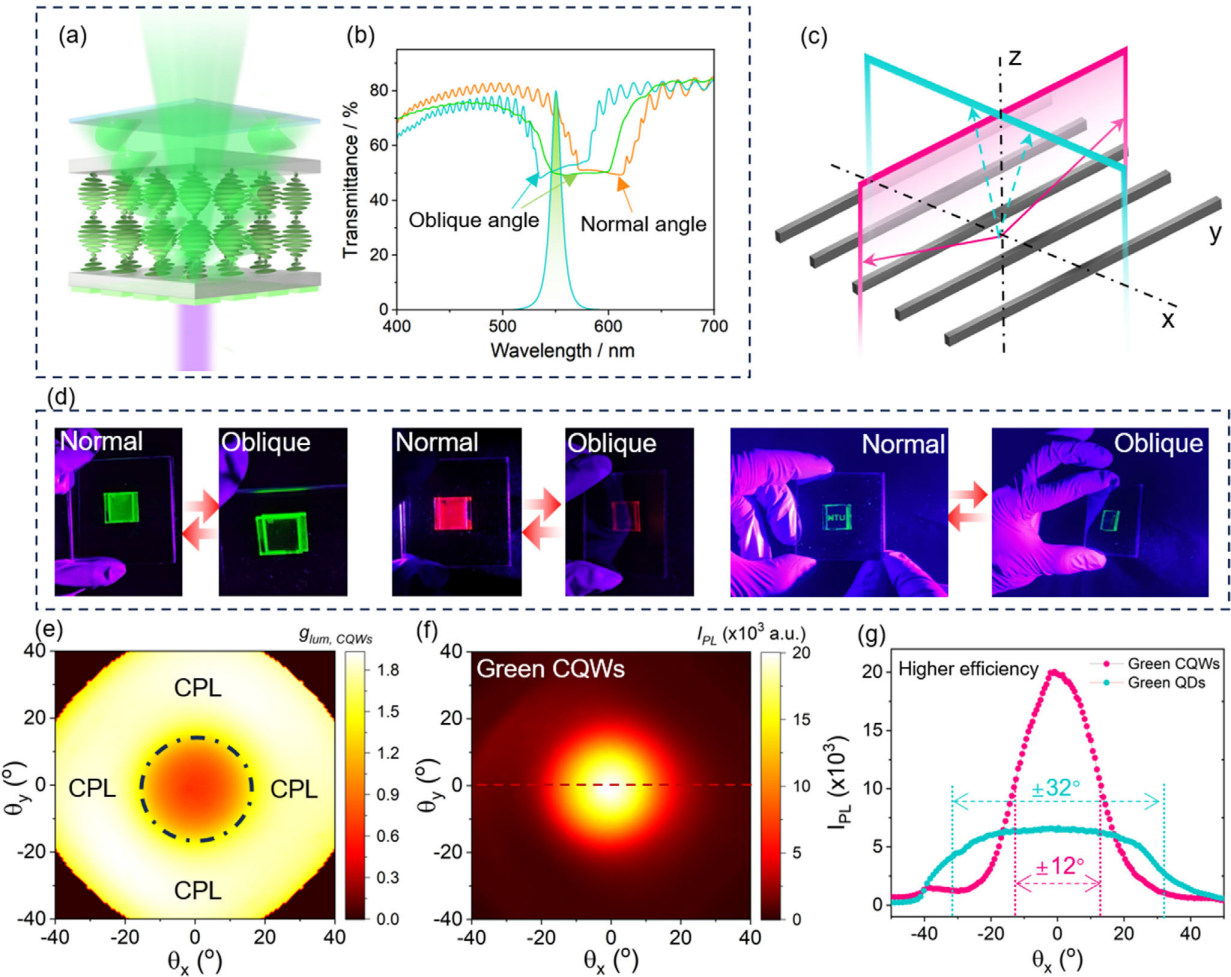
Demonstration of anti‐peep display. a) Schematic illustration of the structure designed for the anti‐peep display. b) Relationship between the reflection band of the CLCs and the PL spectra of the CQWs under normal and oblique viewing angles. c) Schematic of the grating structure in current commercially available anti‐peeping films, which cannot achieve 360‐degree omnidirectional privacy protection. d) Photographs of the fabricated samples observed at normal and oblique angles. Angular distribution of e) *g_lum_* and f) the BFP image of the as‐made anti‐peep CQWs/CLCs sample, characterized from angle‐resolved spectroscopy. g) Cross‐sectional intensity profiles along θ_x_ (dash lines in BFP images) for the CQWs/CLCs and QDs/CLCs samples.

The ultranarrow emission linewidth of CQWs, combined with the angle‐dependent selective reflection properties of LCs, offers multi‐dimensional design flexibility for this integrated system. Here, we demonstrate a display device featuring a 360° omnidirectional anti‐peeping functionality, wherein the displayed pattern is visible only within a narrow front‐facing angular range and becomes invisible at oblique angles, as illustrated in **Figures** **5**a and S25a (Supporting Information). Unlike the goal of achieving a spatially pure CPL as described above, the key mechanism in this design involves precisely positioning the emission of CQWs outside the left edge of the LCs’ reflection band (Figure 5b and Figure S25b, Supporting Information). When paired with a circular polarizer, the emitted light near the normal viewing angle remains non‐circularly polarized and thus passes through the polarizer without being filtered. However, as the viewing angle increases, the CLCs reflection band undergoes a blue shift. Due to the exceptionally narrow FWHM of the CQWs, the PL spectra is rapidly overlapped by the shifted reflection spectrum at larger angles (Figure 5b), resulting in complete extinction of the emission. This characteristic makes the proposed design highly suitable for applications requiring privacy protection, such as anti‐peeping displays. The currently commercialized privacy films utilize a 1D grating structure to achieve privacy protection, resulting in directionally dependent viewing angles, as depicted in Figure 5c. In contrast, our CPL‐based design achieves omnidirectional privacy protection, offering a 360° angular range of effectiveness. To further demonstrate the potential of this approach, we fabricated relevant samples (Figure 5d; Movies S2 and S3, Supporting Information), including fluorescent patterns fabricated based on CQWs using 3D printing techniques (procedures provided in Figure S29, Supporting Information), showcasing the effectiveness of the anti‐peeping feature in practical scenarios. Furthermore, angle‐resolved spectroscopy results shown in Figure 5e–g confirm that for the as‐made green CQWs‐based samples, the CPL is spatially pure at oblique angles, which is critical for achieving a narrow front‐facing viewing angle in line with the design principle. Figure 5f further demonstrates a 50% reduction in light intensity at a narrow angle of ±12°, whereas green QDs‐based samples, due to their broader emission linewidths, achieve a similar intensity drop at a significantly larger angle of ±35° (Figure S26, Supporting Information). Analogous results were observed for red CQWs and QDs samples, as depicted in Figure S28 (Supporting Information). Moreover, the directional emission and ultranarrow linewidth of face‐down CQWs result in enhanced light extraction efficiency in the frontal viewing direction (Figure 5f). In contrast, for QDs‐based samples, a portion of the radiation at normal incidence interacts with the LCs and is filtered by the polarizer due to their broader linewidth. Therefore, the integration of CQWs and CLCs in this design ensures privacy protection without azimuthal angle dependency, while simultaneously improving energy efficiency in the frontal viewing direction.

**Figure 6:**
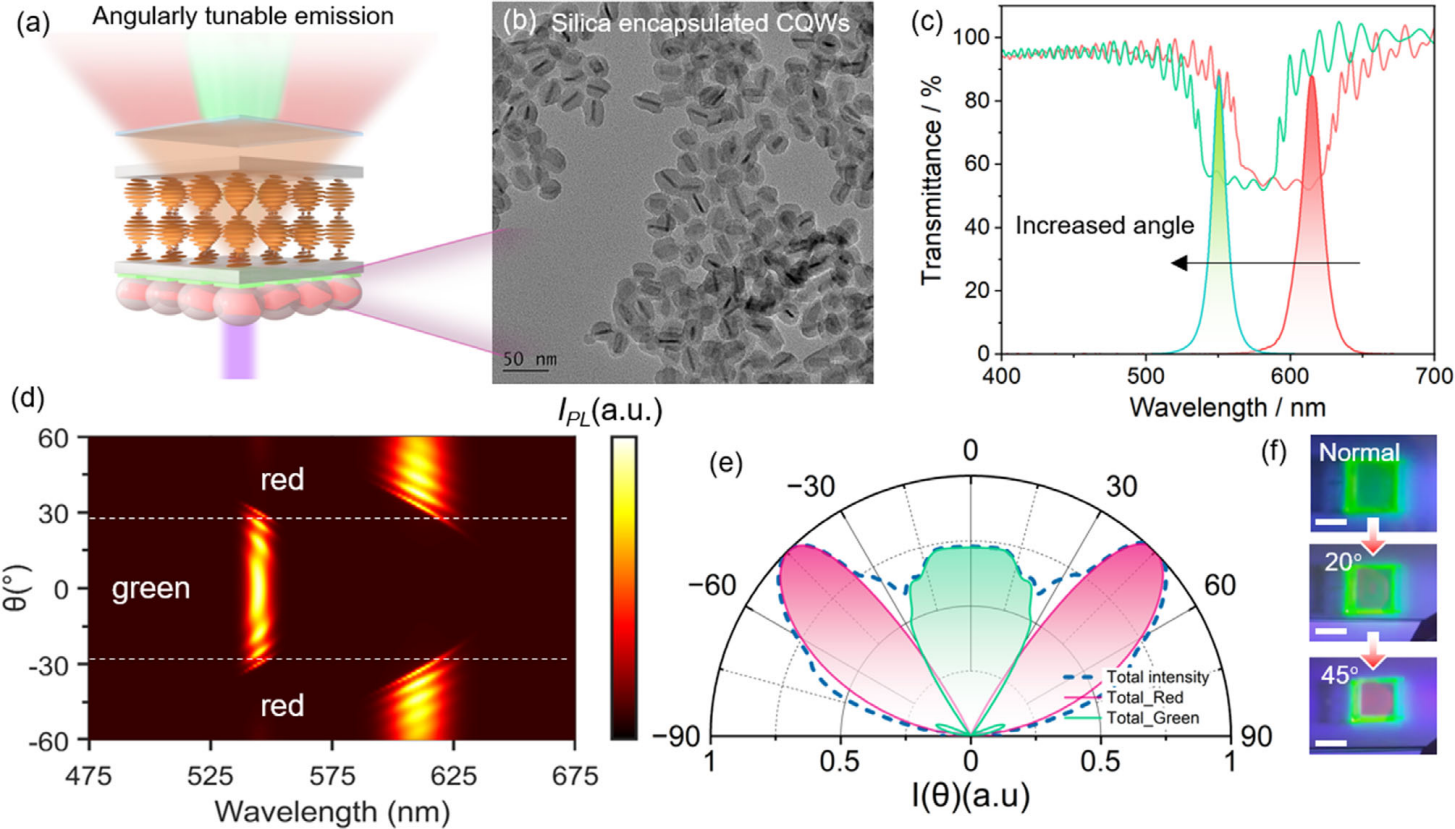
Demonstration of angle‐dependent emission. a) Schematic illustration of the structure designed to achieve angle‐dependent emission. b) TEM image of silica‐encapsulated red‐emitting CQWs. c) Relationship between the reflection band of the CLCs and the PL spectra of the green‐ and red‐emitting CQWs as the viewing angle increases. Simulated d) angle‐dependent PL spectra and e) integrated angle‐dependent PL intensity of the proposed design. f) Photographs of the fabricated sample observed under different viewing angles (Scale bar: 1 cm).

Another design enables angle‐dependent luminescence characteristics by combining green‐emitting CQWs and red‐emitting CQWs within a carefully structured system (**Figure** **6**a). The emission of the green CQWs (centered at 549 nm) was positioned outside the left edge of the LCs' reflection band, while a layer of red‐emitting CQWs was spin‐coated onto the green‐emitting CQWs film. The emission of the red CQWs was strategically located just inside the right edge of the LCs' reflection band, as shown in Figure 6c. At normal viewing angles, the green emission passes through freely, while the red emission is effectively filtered out. As the viewing angle increases, the LCs reflection band undergoes a blue shift, overlapping with and extinguishing the green emission while simultaneously uncovering the red emission. This results in a dynamic, angle‐dependent luminescence, where the system exhibits green light at normal angles and shifts to red at oblique angles (Figure 6d–f; Movie S4, Supporting Information). Here two key issues were addressed in realizing this design: preventing the quenching of green CQWs emission due to Förster resonance energy transfer (FRET) with the red ones, and preserving the integrity of the green CQWs film during the spin‐coating of the red CQWs, given their shared nonpolar surface chemistries. To solve these issues, the red CQWs were encapsulated with a silica shell, increasing the separation between the red and green CQWs by 7 nm (Figure 6b), effectively suppressing FRET. Additionally, the polar hydroxyl groups on the silica surface transformed the nonpolar CQWs into polar ones, allowing their dispersion in ethanol. This orthogonal solvent system prevented damage to the green CQWs film during the spin‐coating process. While angle‐dependent reflection is a well‐documented phenomenon, angle‐dependent luminescence remains scarcely reported. This unique property holds potential for applications in optical anti‐counterfeiting and related fields. This spectrally and angularly selective emission design can also be adapted for soft robotic sensing, where mechanical deformation alters the viewing angle and thereby modulates the observed color output, enabling optical detection of strain or motion.

## 3 Conclusion

In summary, we proposed and demonstrated a bilayered architecture consisting of face‐down self‐assembled CQWs and soft helical superstructures of CLCs with engineered photonic bandgaps. This design enabled highly efficient and spatially pure CPL with an enhanced asymmetry factor of 1.47–1.82 (±0.03) over an expanded viewing angle exceeding ±40°, along with a theoretically calculated 53.3% increase in CPL efficiency. The enhancement of CPL characteristics arises from the synergistic effects of in‐plane oriented TDMs and the narrow emission profiles of CQWs. These factors enable a greater proportion of emitted light to fall within the light escape cone, facilitating efficient CPL conversion through the substantially enhanced spatial coupling between the CQWs' emission and the CLCs' photonic bandgap. These findings were validated through custom‐built angle‐ and wavelength‐resolved spectroscopy, along with optical simulations using the FDTD method. Moreover, the multi‐dimensional design flexibility of this integrated system further facilitated the development of novel 360° omnidirectional anti‐peeping and angular‐dependent luminescent devices. This work lays the groundwork for advancements in CPL‐based photonic technologies through novel design strategies, optical characterization techniques and material platforms.

## Conflict of Interest

The authors declare no conflict of interest.

## Author Contributions

X.L. and T.T.H.D. contributed equally. H.V.D. and X.L. conceived the idea, and H.V.D. supervised the research at all stages. X.L. conducted materials synthesis & characterizations, optical simulations, samples preparations, and device demonstrations. T.T.H.D., S.T.H., and L.D. performed optical characterizations. F.I., E.G.D., and B.C. carried out BFP imaging for self‐assembled CQWs films. S.A. and L.H.W. provided technical supports for TRPL measurements. A.S. helped with CQWs synthesis. X.L. and T.T.H.D. analyzed data and wrote the initial draft of the manuscript. H.V.D., A.I.K., S.T.H., and L.D. revised and finalized the manuscript with inputs from all authors.

## Supporting information



Supporting Information

Supplemental Movie 1

Supplemental Movie 2

Supplemental Movie 3

Supplemental Movie 4

Supplemental Movie 5

## Data Availability

The data that support the findings of this study are available from the corresponding author upon reasonable request.
