# Supplementary material for: Highly Efficient, Spatially Pure Circularly Polarized Luminescence from Bilayer Self‐Assembled Colloidal Quantum Wells and Soft Helical Superstructures
Source: Adv Sci (Weinh). 2025 Aug 13;12(39):e09122. doi: 10.1002/advs.202509122 (PMC12533290; doi:10.1002/advs.202509122)
Supplement: Supplementary file 1 — Supporting Information [file ADVS-12-e09122-s002.docx]

Supporting Information

**Highly Efficient, Spatially Pure Circularly Polarized Luminescence from Bilayer Self-Assembled Colloidal Quantum Wells and Soft Helical Superstructures**

*X. Liang#, T. T. H. Do#, L. Ding, F. Isik,* [*S. Anupam*](https://scholar.google.com/citations?hl=en&user=ZtCiUj8AAAAJ)*, E. G. Durmusoglu, S. Akhil, B. Canimkurbey,* [*L. H. Wong*](https://onlinelibrary.wiley.com/authored-by/Wong/Lydia+H.)*, S. T. Ha, A. I. Kuznetsov, and H. V. Demir**

**Table of contents**

Section 1. Additional experimental methods.

Section 2. Additional information for CLCs samples.

Section 3. Additional information for BFP pattern calculations.

Section 4. Additional characterizations for CQWs and QDs.

Section 5. Additional optical characterizations.

Section 6. Additional optical simulations.

Section 7. Additional information for demonstrations.

**Section 1. Experimental methods.**

***Chemicals***

Cadmium oxide (CdO, 99.9%), cadmium acetate dihydrate (Cd(OAc)_2_·2H_2_O, >98%), cadmium nitrate tetrahydrate (Cd(NO_3_)_2_·4H_2_O, 99.997%, trace metals basis), sodium myristate (≥99%), selenium (Se, 99.99%, trace metals basis), sulfur (S, 99,998%, trace metals basis), zinc acetate (Zn(OAc)_2_, 99.99%, trace metals basis), zinc acetate dihydrate (Zn(OAc)_2_·2H_2_O, 99.999%, trace metal basis), 1-octanethiol (≥98.5%), myristate acid (MA, 99%), IGEPAL CO-520 (average M_n_ 441), tetraethyl orthosilicate (TEOS, ≥99%), ammonium hydroxide ([≥99.99% trace metals basis](https://www.sigmaaldrich.com/SG/en/product/sigald/338818)), 1-octadecene (ODE, technical-grade), oleic acid (OA, 90%), oleylamine (OAm, 70%), and trioctylphoshine (TOP, 90%) were purchased from Sigma-Aldrich. Methanol, hexane, cyclohexane, toluene, and ethanol were obtained from Merck Millipore and used without any further purification.

***Preparation of cadmium myristate precursors***

Cadmium myristate (Cd(Myr)_2_) was prepared following a modified method.^[1]^ First, 1.23 g of Cd(NO_3_)_2_·4H_2_O and 3.13 g of sodium myristate were dissolved in 40 mL and 250 mL methanol, respectively. The solutions were combined, stirred vigorously for 1 hour, and Cd(Myr)_2_ precipitated. The solid was separated by centrifugation, washed with methanol at least three times to remove impurities, and dried under vacuum at 50 °C overnight.

***Preparation of 0.1 M S-ODE precursors***

0.1 M S-ODE solution was prepared by dissolving 32 mg of S in 10 mL of ODE with ultrasonication for 30 minutes.

***Synthesis of CdSe/CdSe_x_S_1-x_ core/gradient crown CQWs***

The core/gradient crown CQWs were synthesized *via* a one-pot method, where crown precursors were added during CdSe core growth. As Se was depleted and S-precursors were continuously supplied, a CdSe_x_S_1-x_ gradient crown formed around the CdSe core. Using this strategy, the PL peak could be tuned between 463 nm and 549 nm by varying the CdSe core thickness (3, 4, or 5 monolayers). For 4 ML cores, 170 mg of Cd(Myr)_2_, 12 mg of Se powder, and 15 mL of ODE were loaded into a 50 mL three-neck flask, degassed for 30 min at room temperature, then purged with N_2_ and heated to 230 °C. At 195 °C, 80 mg of Cd(OAc)_2_·2H_2_O was quickly added, initiating the transition to a CdS gradient crown through the slow addition of 1.5 mL of 0.1 M S-ODE at 240 °C over 8 min. The mixture was cooled to room temperature, with 1 mL of OA added at 150 °C and 10 mL of hexane at 60 °C. The CQWs were precipitated using 5 mL of ethanol, centrifuged at 4,000 rpm for 10 min, washed, and redispersed in 5 mL of hexane. For 3 ML or 5 ML cores, a similar approach was applied, following specific CdSe core growth methods outlined in the referenced literature.^[2]^

***Synthesis of 4ML CdSe cores***

CdSe 4ML cores were synthesized following a modified protocol.^[3]^ In a 50 mL three-neck flask, 170 mg of Cd(Myr)_2_, 12 mg of Se, and 15 mL of ODE were combined, degassed for 20 min, purged with N_2_, and heated to 240 °C. At ~195 °C, 80 mg of Cd(OAc)_2_·2H_2_O was added rapidly. After 10 min growth, the solution was cooled to room temperature, with 1 mL of OA added at 150 °C and 10 mL of hexane at 60 °C. CdSe 4ML cores were precipitated with 5 mL ethanol, separated by centrifugation (4,000 rpm, 10 min), washed, and redispersed in 5 mL hexane.

***Shell growth for synthesizing core@shell or core/gradient crown@shell CQWs***

To achieve red-shifted emission and full visible spectrum coverage, the CQWs thickness was increased *via* a hot-injection shell growth method to reduce the bandgap. Typically, 0.4 mmol of Cd(OAc)_2_·2H_2_O / Zn(OAc)_2_, 1 mL of OA, and 10 mL of ODE were combined in a 50 mL three-neck flask, degassed under vacuum for 20 min, and then purged with N_2_. The mixture was heated to 200 °C for 30 min to form a clear solution before being cooled to 60 °C. At this point, 1 mL of CdSe/CdSe_x_S_1-x_ 4ML core/gradient crown CQWs or CdSe 4ML cores in hexane was added, followed by 45 min of vacuum degassing to remove residual hexane. After switching to N_2_ flow, the solution was reheated to 300 °C, and 1 mL of degassed OAm was added at 90 °C. A 0.1 M 1-octanethiol solution in ODE (4 mL) was injected as the S-source at 8 mL/h using a syringe pump, starting at 165 °C and continuing until all the precursor was consumed. The reaction mixture was then annealed for 40 min before being quenched in a water bath. At 60 °C, 10 mL of hexane was added, and the CQWs were precipitated with 10 mL of ethanol, washed twice, and redispersed in hexane or toluene. By adjusting the Cd/Zn precursor molar ratio, the shell composition and emission peaks could be tuned within 600–675 nm.

***Synthesis of green CdSe@ZnS QDs***

Green QDs emitting at 535 nm were synthesized using a modified literature method.^[4]^ In a 100 mL three-neck flask, 0.4 mmol CdO, 4 mmol Zn(Ac)_2_, 5 mL OA, and 15 mL ODE were mixed and heated to 90 °C under vacuum for 30 min. The mixture was then heated to 310 °C under N_2_, and 3 mL TOP containing 0.2 mmol Se and 4 mmol S was swiftly injected. After 10 min, the solution was cooled to room temperature in air. QDs were purified by adding ethanol until turbidity, followed by centrifugation at 6,000 rpm for 5 min; this step was repeated twice. Purified QDs (80% quantum yield) were dispersed in toluene for later use.

***Synthesis of silica-encapsulated CQWs***

Silica encapsulation of bare CQWs followed a modified method from prior research.^[5]^ At room temperature, 10 mL cyclohexane and 1.2 g IGEPAL CO-520 were mixed. Around 30 mg of CQWs, precipitated from hexane and redispersed in 300 μL octane, were added to the mixture, followed by 120 μL TEOS under continuous stirring. After 20 min, 300 μL ammonium hydroxide was introduced to initiate the reaction, which proceeded for 48 h. Silica-coated CQWs were separated by adding 2 mL ethanol and centrifugated at 6,000 rpm for 3 min. The product was washed and redispersed in 10 mL ethanol for further use.

***Fabrication of planar-oriented CLCs cells***

A certain amount of nematic LCs (SLC1717, purchased from ShijiazhuangChengzhi Yonghua Display Materials Co., Ltd.) and the chiral compound (S1011, purchased from Merck Co., Ltd.) were mixed homogeneously under heated conditions in a specific ratio. Here S1011 was selected due to its high helical twisting power compared to other commonly used chiral dopants such as S811.^[6]^ This allows for a broad liquid-crystalline working temperature range without significantly affecting the clearing point of SLC1717, thereby ensuring thermally stable reflection properties and enabling precise control over the coupling between CQWs emission and the CLCs reflection band. Further details are provided in **Figures S3** and **S4** of the Supporting Information and in Supplementary Movie S5. The mixture was introduced into a LCs cell with a polyimide planar alignment treatment on the inner surface *via* capillary action. The thickness of the LCs cell was controlled at 12 μm using spacers. The LCs cell was heated on a hot stage until the LCs reached a clear state and then cooled to room temperature to obtain a planar-aligned CLCs layer.

***Self-assembly of CQWs***

The prepared CLCs cell was placed inside an 80 mm diameter Teflon container filled with acetonitrile (ACN) as the subphase. Subsequently, a 20 µL hexane solution of CQWs was carefully added to the ACN from the edge of the container. Upon contact, the CQWs spread across the surface of the ACN subphase. After the hexane evaporated, a uniform, face-down CQWs film was formed on the subphase surface. To transfer this film onto the surface of the CLCs cell, a peristaltic pump was used to gradually remove the ACN from the bottom of the container, allowing the film to settle gently onto the cell surface. To ensure uniformity, the height of the ACN above the cell was minimized to reduce disturbances in the assembled film during the drainage process. Additionally, a silicone oil surfactant was used to compress the monolayer further, reducing the risk of cracks or voids caused by capillary forces from the container walls during deposition.

***Fabrication of the anti-peep sample***

For CQWs with a fixed emission wavelength profile (5 monolayer CdSe/CdSe_x_S_1-x_, with an emission peak at 549 nm, used as the emitter in this case), the left edge of the reflection band of the CLCs was adjusted to lie just beyond the right side of the CQWs' emission band by tuning the concentration of S1011. The preparation methods for the CLCs cell and the self-assembled CQWs were consistent with those described above.

***Fabrication of the angle-dependent emission sample***

The emission profile of the CQWs and the reflection band of the CLCs were regulated as described in the main text. To achieve switching between green (549 nm) and red (620 nm) emission, the left edge of the CLCs reflection band was adjusted to lie just beyond the right side of the CQWs' emission band. After self-assembling the green CQWs, red-emitting CQWs with an emission peak around 620 nm were selected, as their emission profile could be fully covered by the right edge of the CLCs reflection band. These red CQWs were encapsulated in a silica shell, which served as a spacer to prevent direct contact with the green CQWs while also converting their nonpolar surface chemistry to a polar one. The red CQWs were then dispersed in ethanol and spin-coated onto the self-assembled green CQWs layer.

***Optical setup for the CPL characterization***

The CPL was characterized by a customized angle-resolved spectrally-resolved spectroscopy using an optical microscope in an inverted configuration (Nikon Ti-U). The spectrometer (Andor SR-303i) comprised of a single grating (150 grooves/mm) and a charged-coupled detector (Andor Newton 971). The sample was illuminated by the blue light filtered from a mercury lamp focused onto the sample through a high-numerical-aperture objective (100×, NA=0.8). The emission was detected using the same objective, guided through a lens system for resolving angular information. The entrance slit was opened fully for the back-focal-plane imaging, while it was closed to 100 μm for angle-resolved PL spectra. A quarter-waveplate and a linear polarizer were placed on the detection path to detect left-handed $(I_{L})$ and right-handed $\left( I_{R} \right)$ polarized PL signals. The asymmetric factor $g_{lum}$ was calculated from $I_{L}$ and $I_{R}$.

***Measurement of the absorption, transmittance and PL spectra***

The UV-Vis absorption /transmittance and PL spectra of samples were measured using a Shimadzu UV-1800 and a Shimadzu RF-5301 PC spectrometers, respectively.

***TEM characterization***

The morphologies of both the CQWs and the silica-encapsulated CQWs were characterized using a JEOL 2100F transmission electron microscope, operated at 200 kV.

***SEM characterization***

The surface morphology of the self-assembled samples was examined using a JEOL JSM-7600F scanning electron microscope, operated at an accelerating voltage of 1 kV.

***Quantum yield and florescent lifetime measurement***

Quantum yields were measured using a 405 nm laser within an integrating sphere, with data collected via an Ocean Optics S4000 spectrometer. For time-resolved photoluminescence (TRPL), a TCSPC system (PicoHarp 300) with 4 ps resolution was used, featuring an 80 MHz pulsed laser. The setup included a PDL-800 driver for a 375 nm (3.31 eV) picosecond laser and a Hamamatsu H5783 photomultiplier tube for resolving picosecond lifetimes.

***BFP imaging for face-down self-assembled CQWs and spin-coated QDs***

Self-assembled CQWs and spin-coated QDs on quartz substrates were excited by a 400 nm laser. The resulting fluorescence was collected by an inverted optical microscope (Nikon eclipse Ti-U) with a high numerical aperture (NA) objective lens of 1.2 and immersion oil and directed to the Fourier plane. It passed through a 4-f system followed by a 550 nm long-pass filter and a polarizer before reaching the charge-coupled device (CCD) camera (Thorlabs) for imaging, as illustrated in **Figure S6**.

**Section 2. Additional information for CLCs samples.**


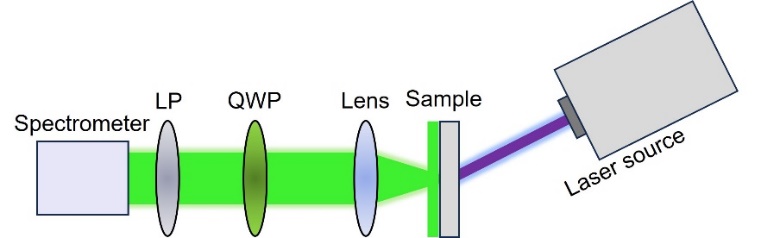


**Figure S1.** Schematic illustration of the conventional setup for characterizing CPL performance. The polarized light emitted by the sample is converted to linearly polarized light using a quarter-wave plate (QWP) and subsequently analyzed by a spectrometer after passing through a linear polarizer (LP).

**
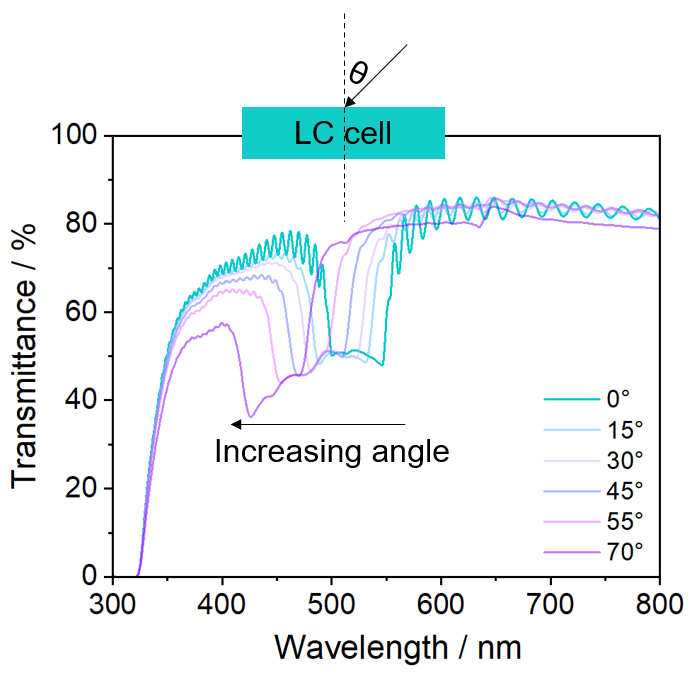
**

**Figure S2.** Angle-dependent reflection of planar-oriented CLCs with a central wavelength of 535 nm.

**Table S1.** Physical parameters of the nematic LCs (SLC-1717) used in this study.


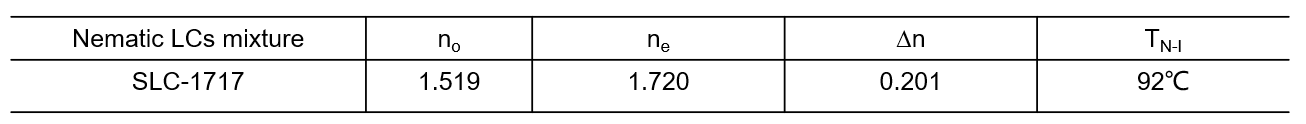


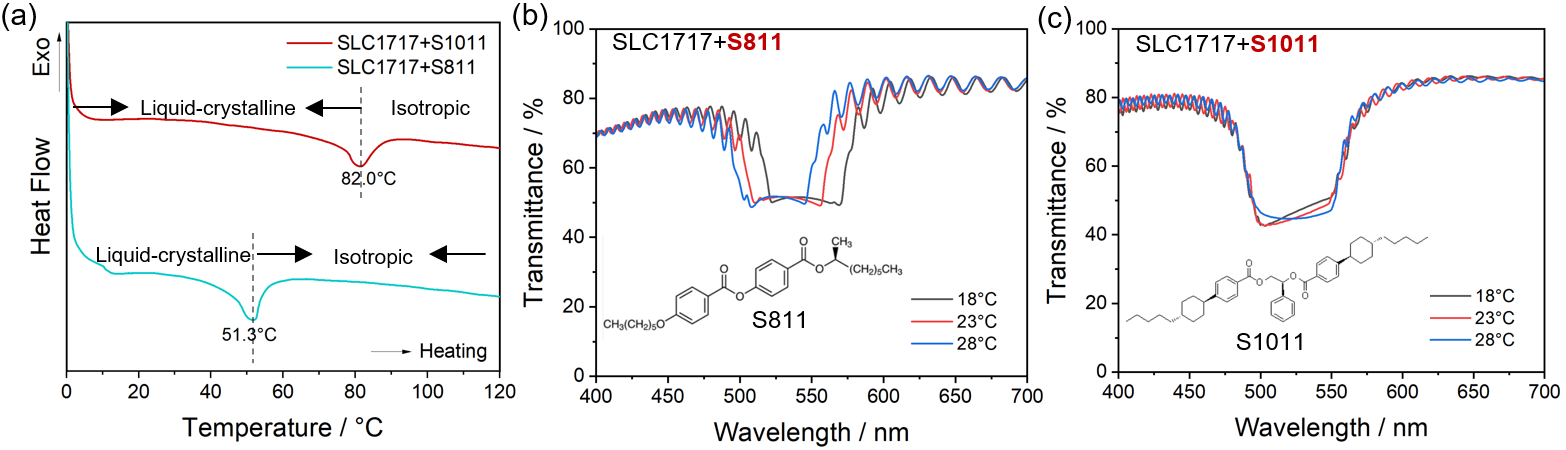


**Figure S3.** (a) DSC characterization of CLCs doping with S811 (green) and S1011 (red), respectively. The clear point of the commercial SLC1717 is ~92°C. (b) Temperarture-dependent reflection spectra of planar aligned CLCs using (b) S811 and (c) S1011, respectively. The chemical structure of S811 and S1011 are inserted in (b) and (c), respectively.


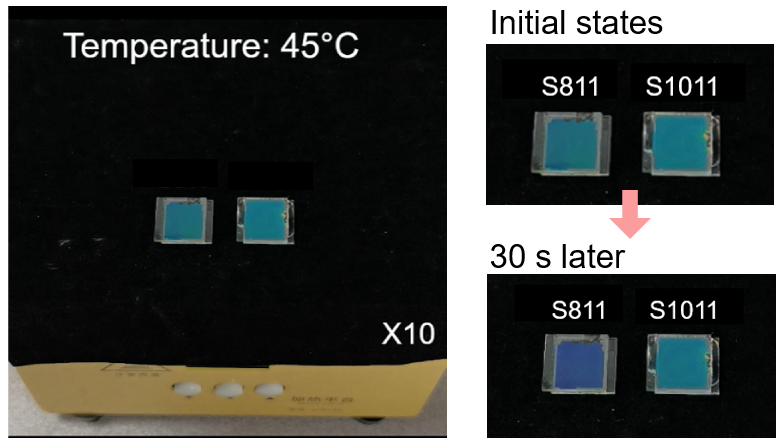


**Figure S4.** Photographs showing the thermal response of the reflection spectra for samples prepared with S811 and S1011, respectively.

S**ection 3. Additional information for BFP pattern calculations.**


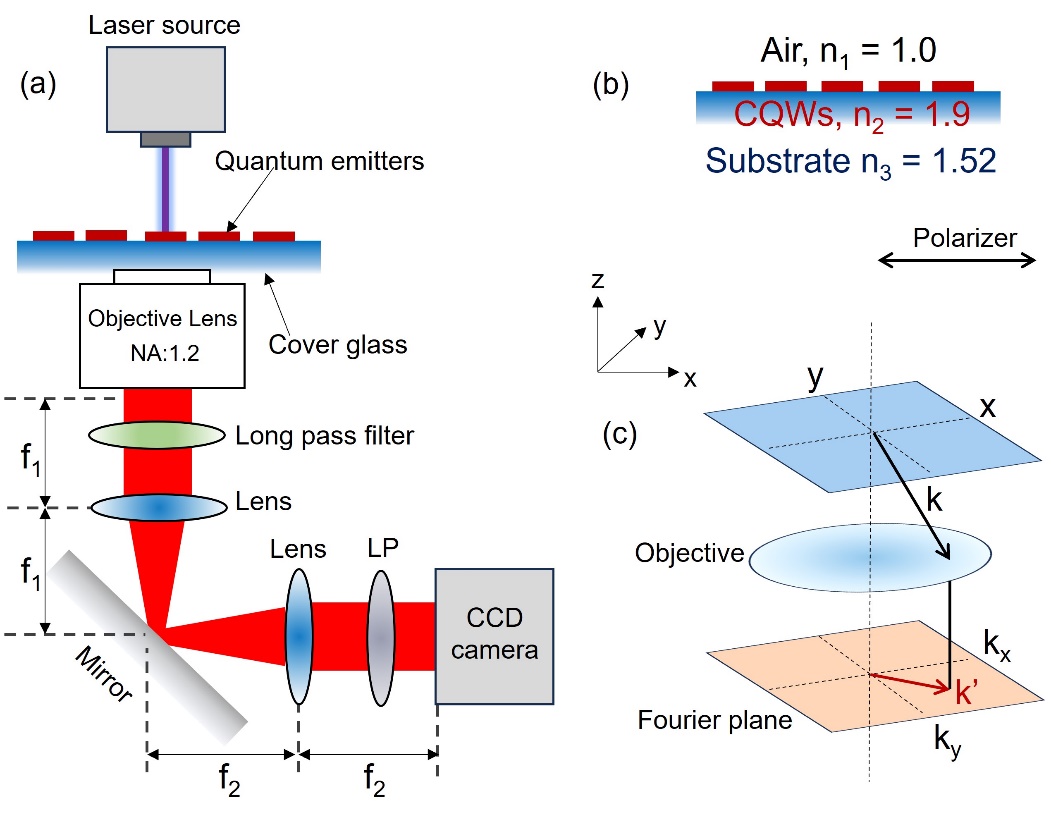


**Figure S5.** (a) Schematic of the optical setup for BFP imaging. (b) Schematic representation and parameters of the three-layered structure used in BFP simulations. (c) Emission configuration with the coordinate system used for simulations.


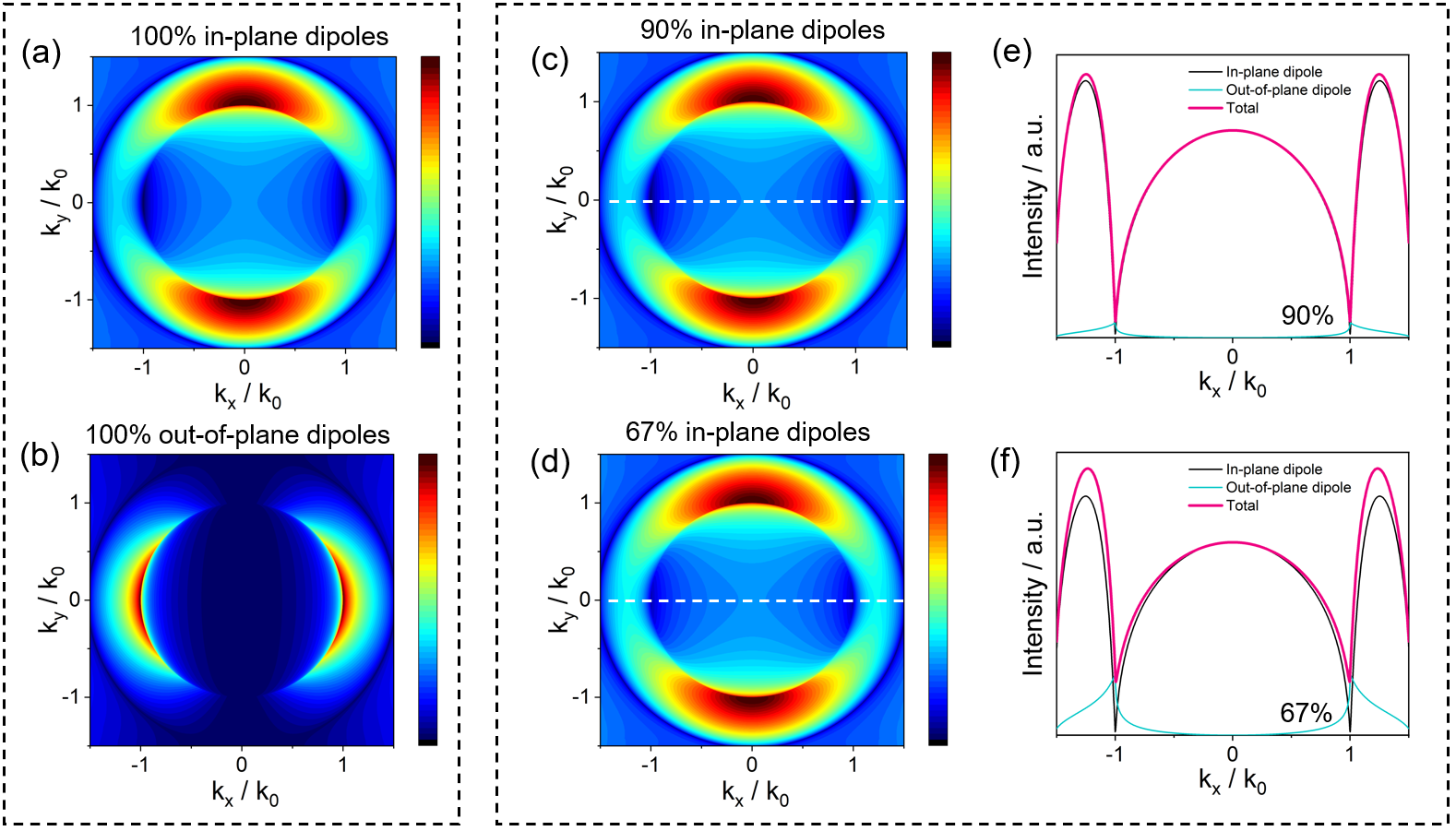


**Figure S6.** Simulated BFP patterns with (a) 100% in-plane dipoles, (b) 100% out-of-plane dipoles, (c) 90% in-plane dipoles, and (d) 67% in-plane dipoles. Corresponding intensity profiles along the white dashed lines are presented in (e) and (f), respectively, demonstrating the varying dipole contributions.


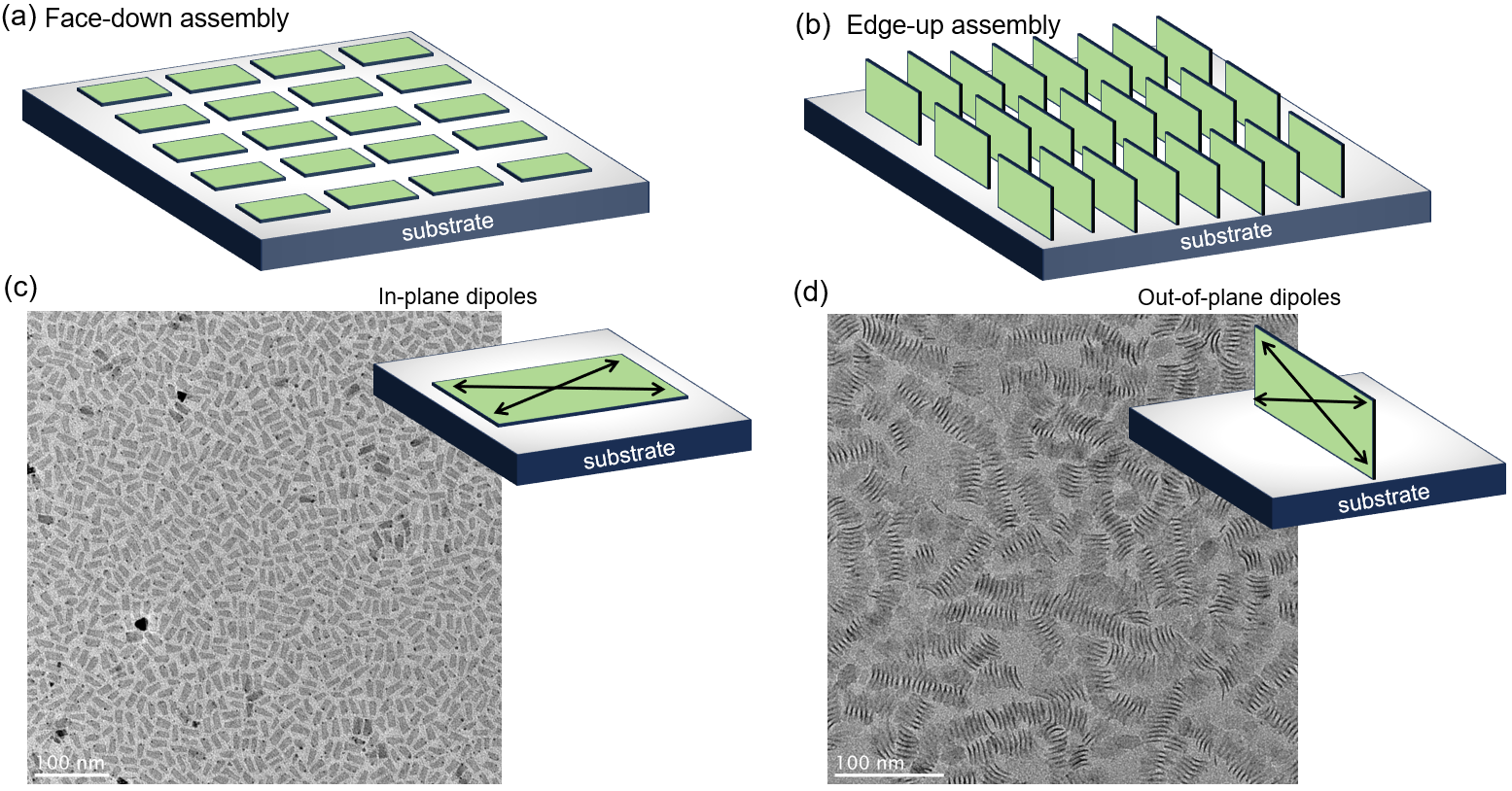


**Figure S7.** Schematics of (a) face-down and (b) edge-up self-assembly of CQWs. Representative TEM images of (c) face-down and (d) edge-up self-assembled CQWs, respectively. In the face-down configuration, the CQWs exhibit 100% in-plane dipole orientation relative to the substrate, whereas in the edge-up configuration, the CQWs exhibit 100% out-of-plane dipole orientation.

**Section 4. Additional characterizations for CQWs and QDs.**


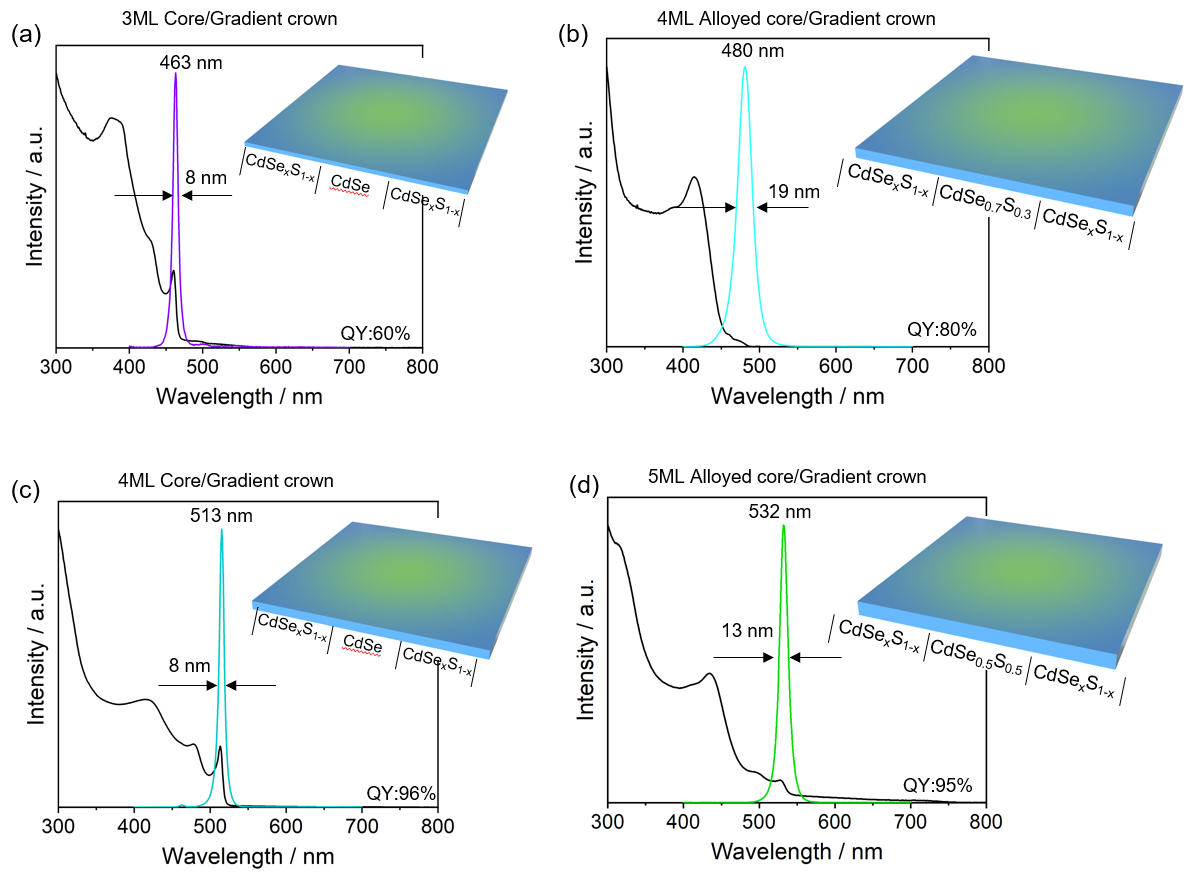


**Figure S8.** Absorption and PL spectra of representative core/gradient crown CQWs with emission peaks at (a) 463 nm (3ML core/gradient crown), (b) 480 nm (4ML alloyed core/gradient crown), (c) 513 nm (4ML core/gradient crown) and (d) 532 nm (5ML alloyed core/gradient crown). Insets show schematic diagrams of the structures and compositions of the respective CQWs.


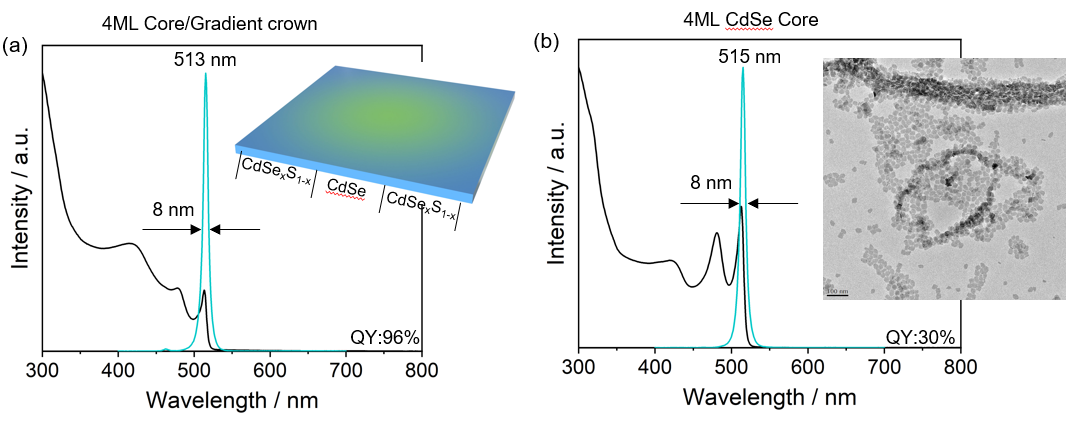


**Figure S9.** (a) 4ML CdSe/CdSe_x_S_1-x_ CQWs and (b) 4ML CdSe CQWs used for subsequent shell growth. The inset in (b) displays a TEM image of the CdSe CQWs.


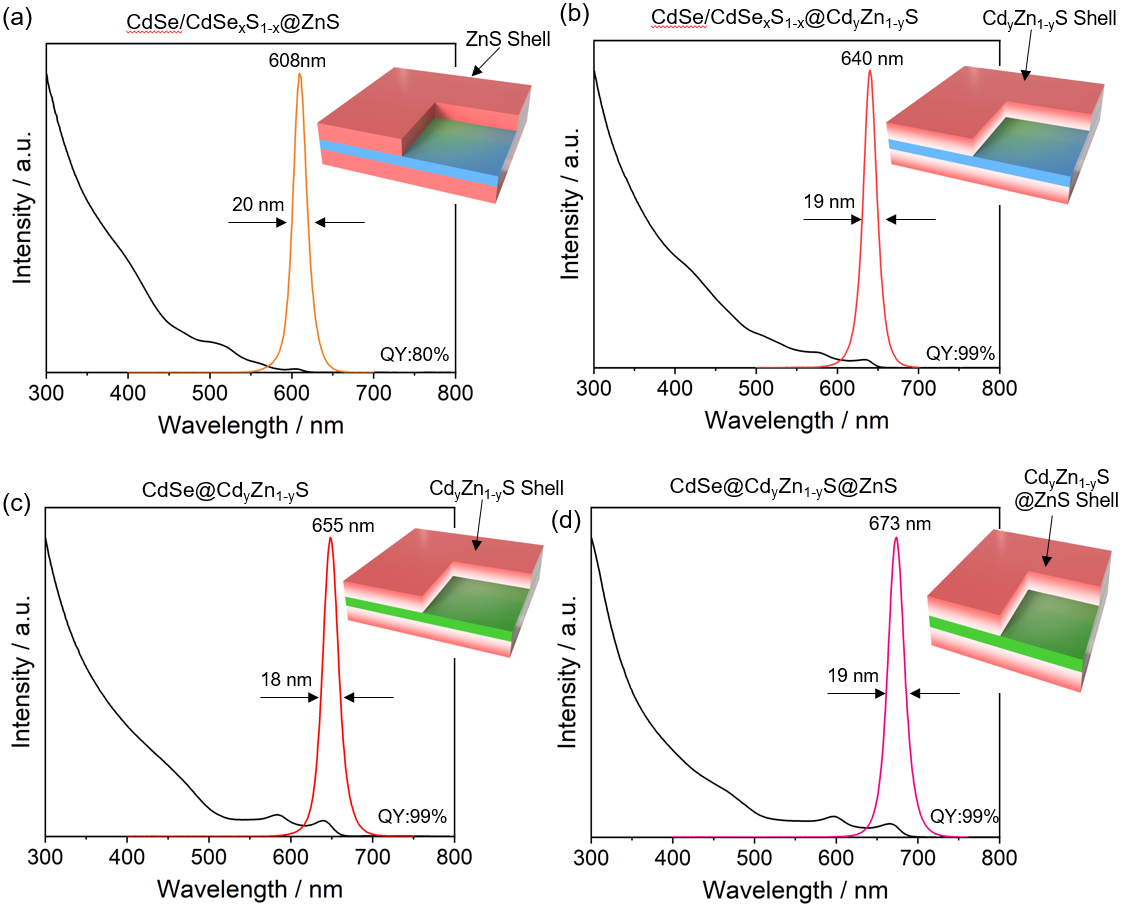
.

**Figure S10.** Absorption and PL spectra of representative core/gradient crown@shell CQWs with peak emission at (a) 601 nm, (b) 640 nm, (c) 655 nm and (d) 673 nm, respectively. Insets illustrate the structures and shell compositions of the respective CQWs.


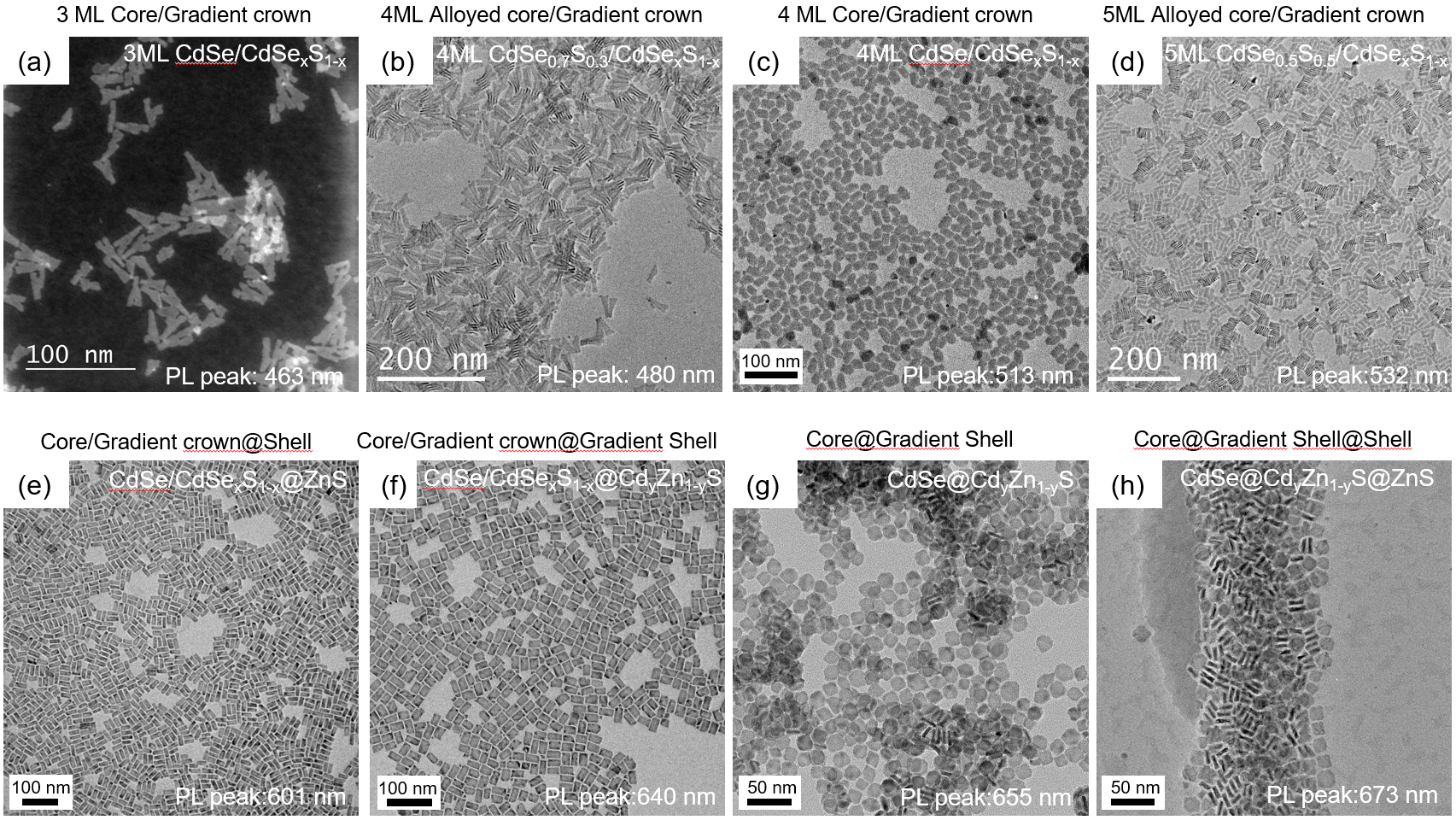


**Figure S11.** TEM images of CQWs with different structures and emission wavelengths.


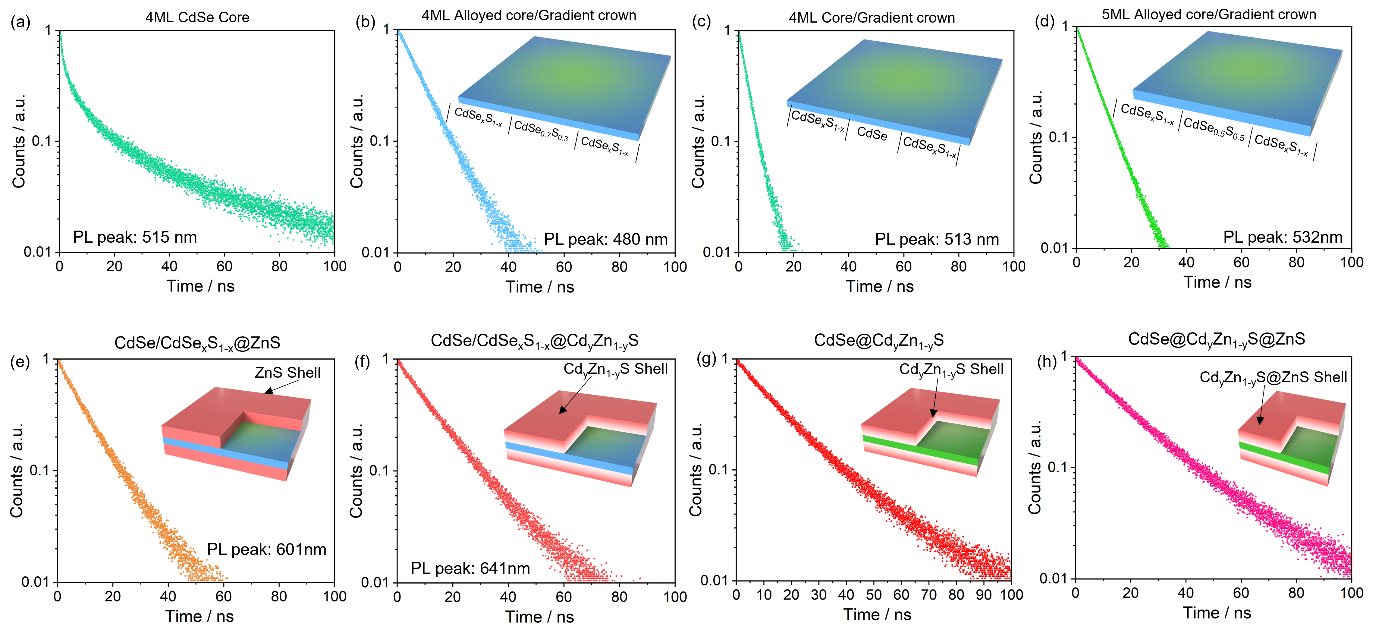


**Figure S12.** Fluorescence lifetime measurements of CQWs with various architectures: (a) conventional 4ML CdSe core CQWs with a QY of 30%, and (b–h) synthesized CQWs with near mono-exponential decay profiles, consistent with their high QYs.


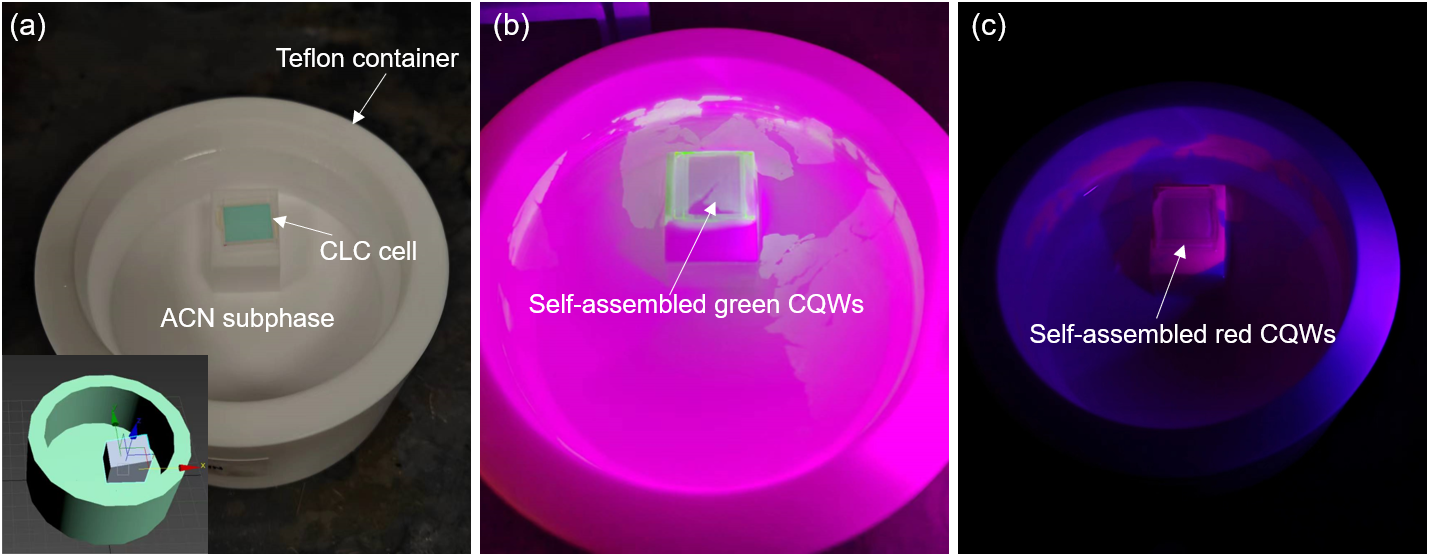


**Figure S13.** Setup for the self-assembly experiment.


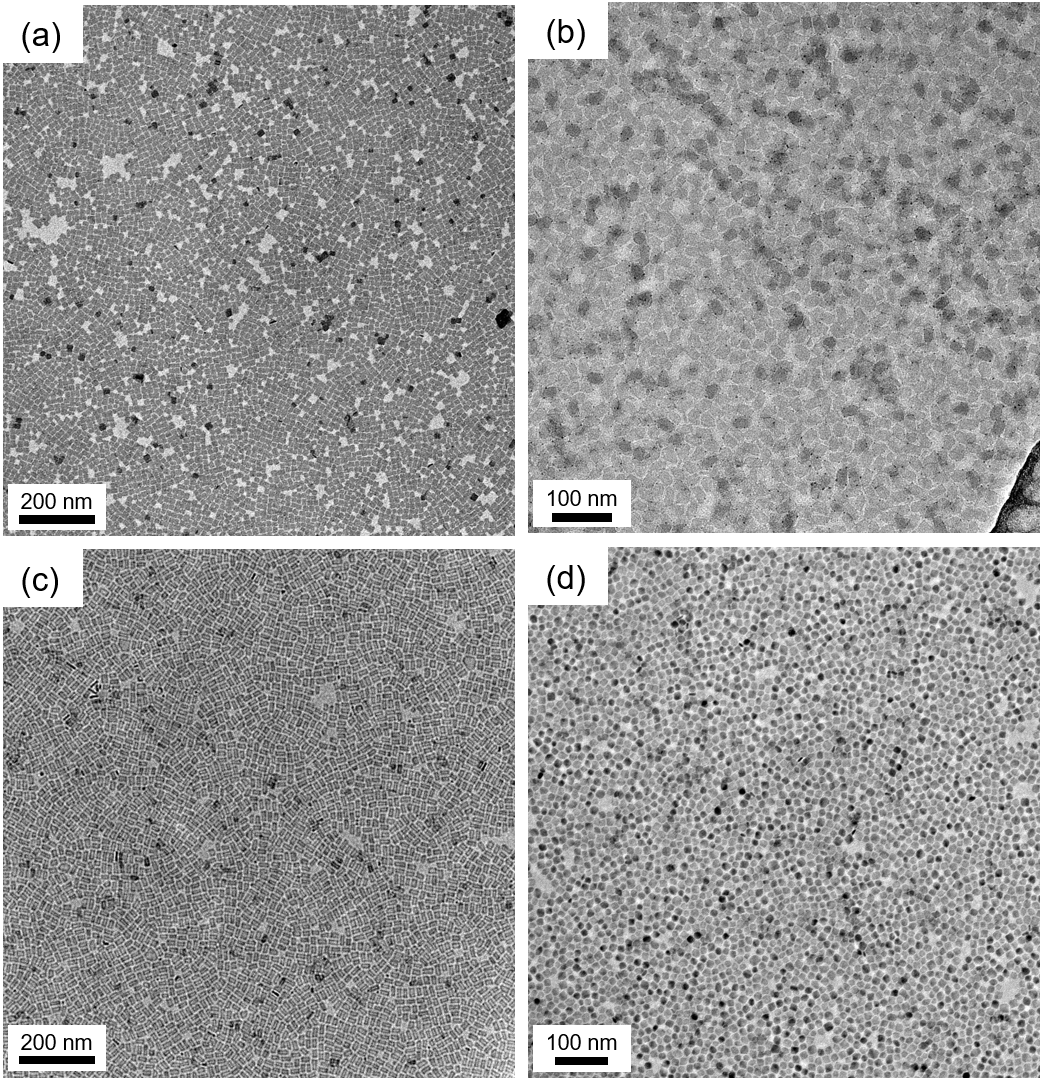


**Figure S14.** Self-assembly of CQWs with different architectures.


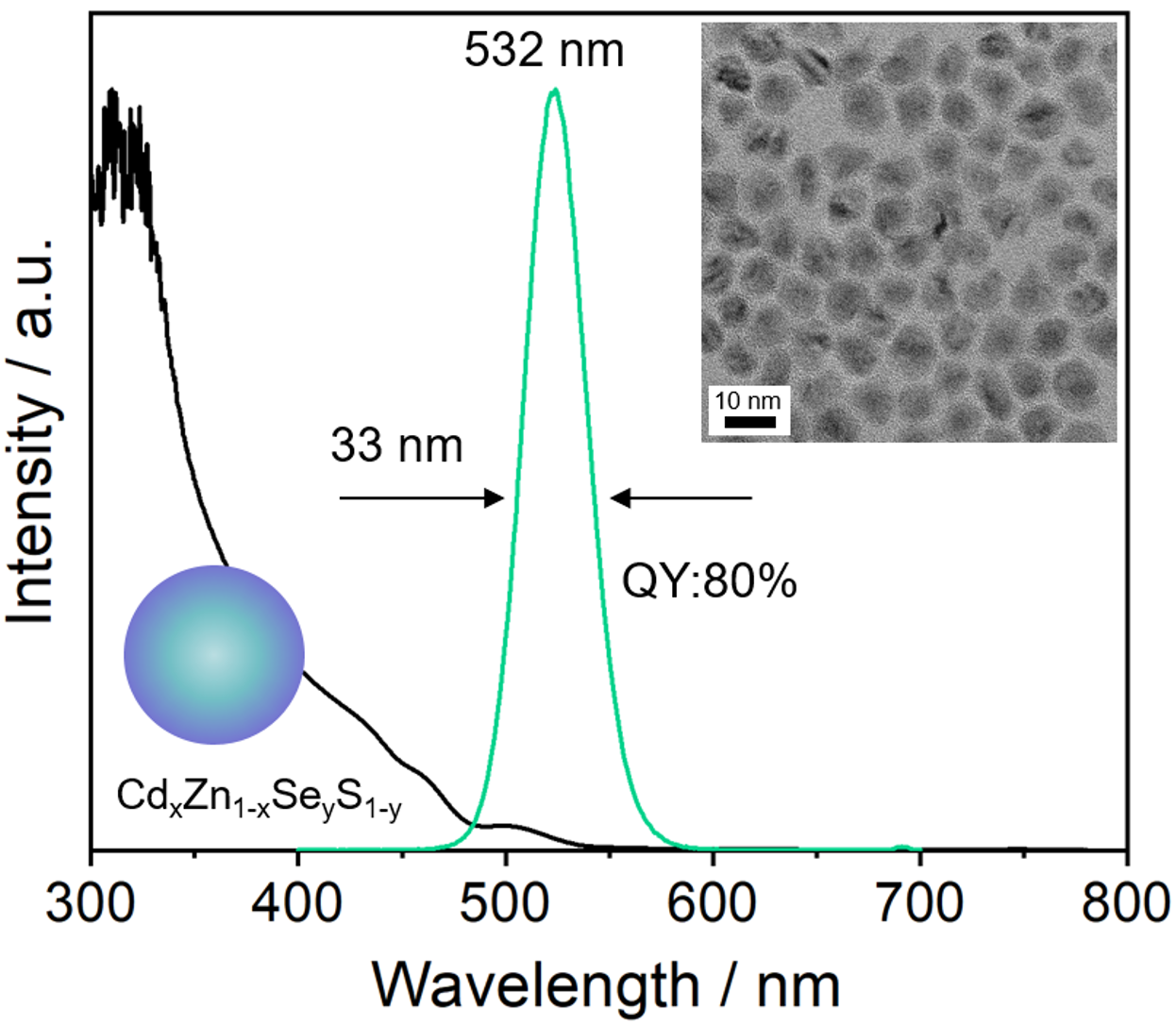


**Figure S15.** Absorption and PL spectra of the green QDs used in this study for comparison. Insets show the TEM image of the QDs.

**Section 5. Additional optical characterizations.**


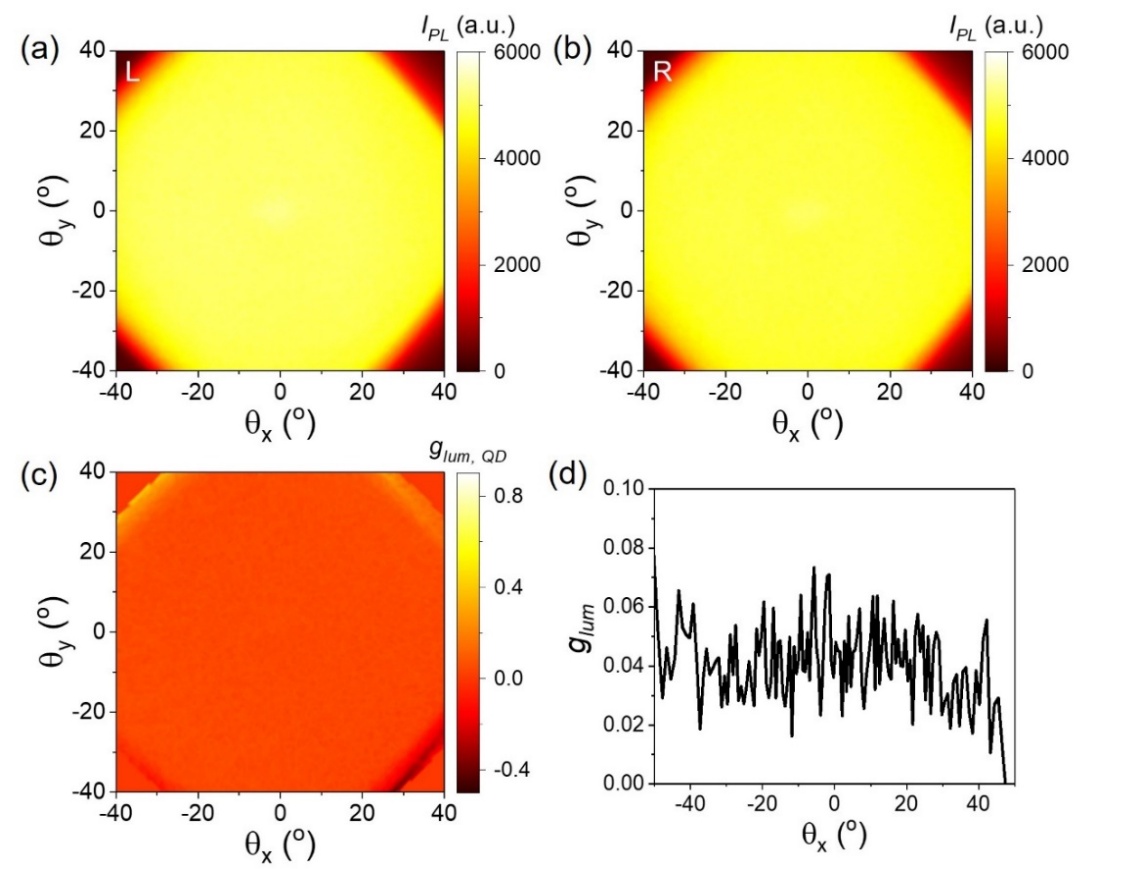


**Figure S16.** Angle-resolved PL imaging of a reference sample of QDs without CLCs.


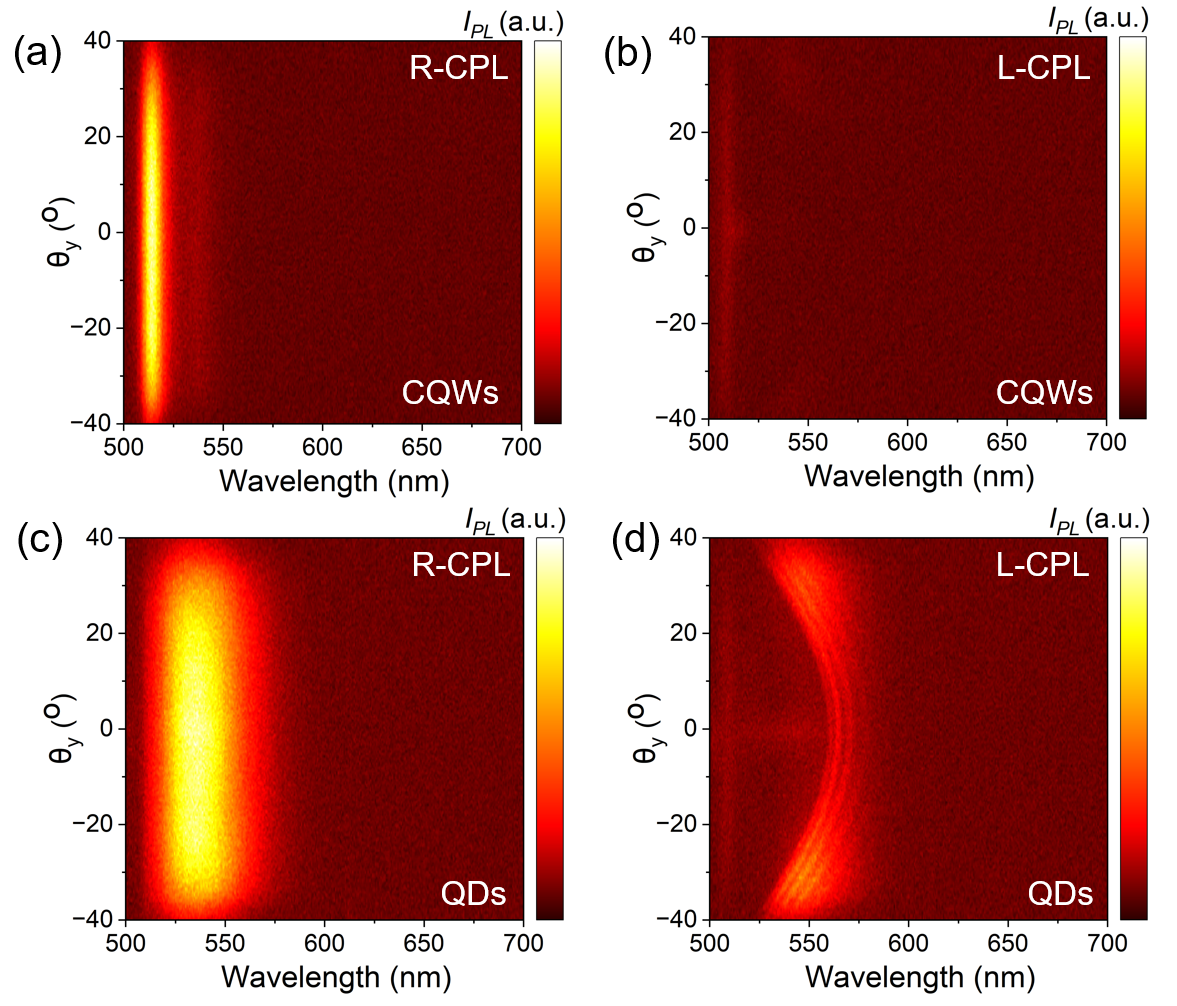


**Figure S17.** Angular- and wavelength-resolved PL results for CQWs/CLCs filtered through (a) right-handed and (b) left-handed circular polarizers, respectively. Angular- and wavelength-resolved PL results for QDs/CLCs filtered through (c) right-handed and (d) left-handed circular polarizers, respectively.


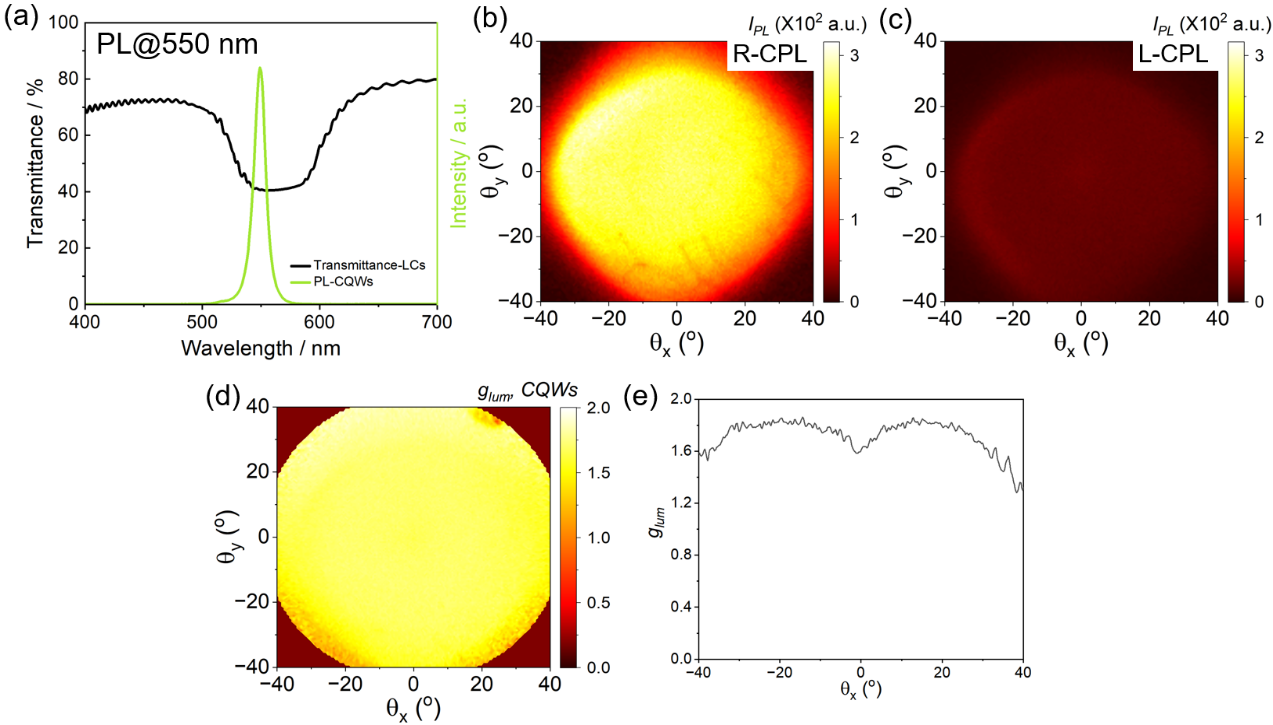


**Figure S18.** (a) Alignment of the LCs reflection band with the PL spectra of CQWs emitting at 550 nm. BFP images of the CQWs/CLCs sample, filtered through right-handed (b) and left-handed (c) circular polarizers, respectively. (d) Calculated *g_lum_* for the CQWs/CLCs sample. (e) θ_x_-dependent *g_lum_* for the sample.


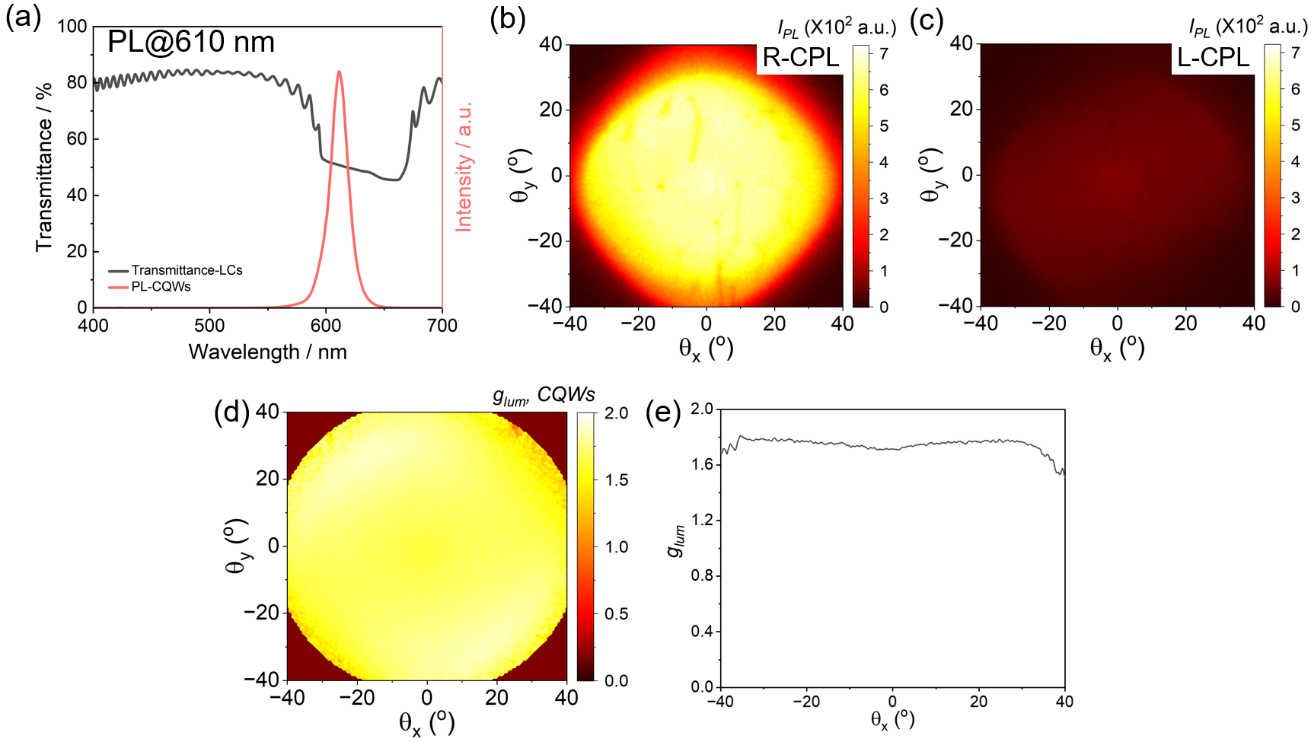


**Figure S19.** (a) Alignment of the LCs reflection band with the PL spectra of CQWs emitting at 610 nm. BFP images of the CQWs/CLCs sample, filtered through right-handed (b) and left-handed (c) circular polarizers, respectively. (d) Calculated *g_lum_* for the CQWs/CLCs sample. (e) θ_x_-dependent *g_lum_* for the sample.

**Section 6. Additional optical simulations.**


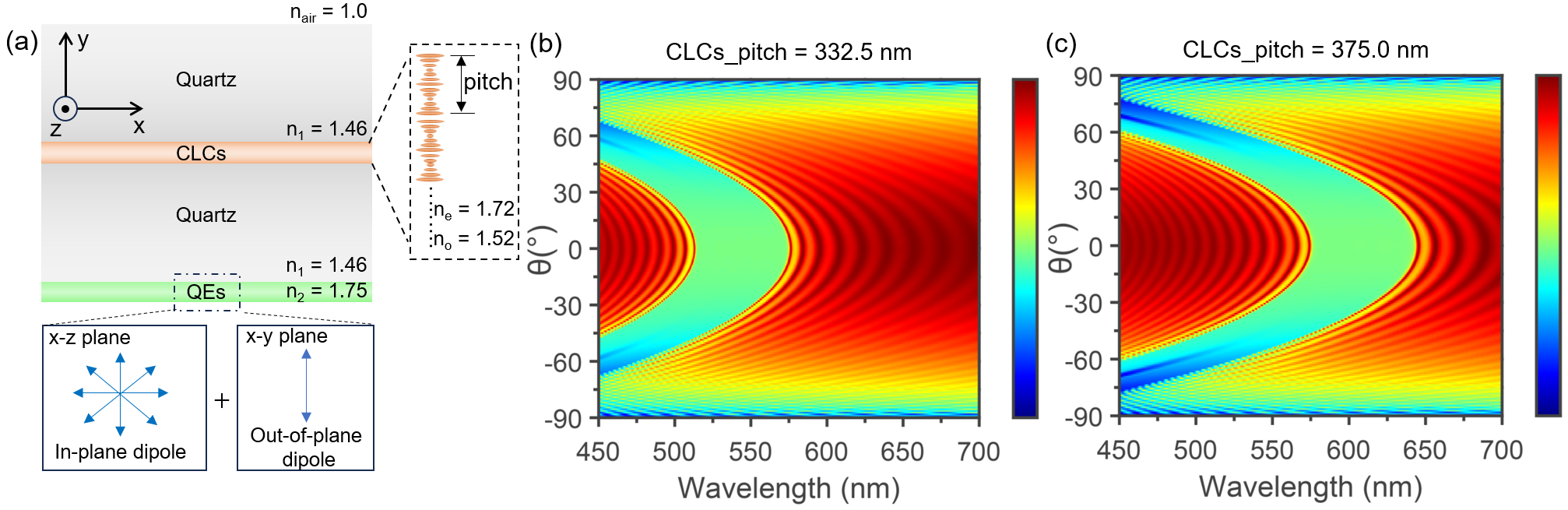


**Figure S20.** (a) Schematic representation of the QE/CLC model used in the simulation. The dielectric anisotropy of the CLCs was introduced through their permittivity tensor. A dipole source was used as the light source and positioned at the center of the QEs layer to simulate the emission properties. Simulated reflection band spectrum of the CLCs with a pitch of (b) 332.5 nm and (c) 375 nm, respectively.

Optical simulations were carried out using the Finite-Difference Time-Domain (FDTD) method. The simulation model and associated parameters are illustrated in **Figure S20a**. In these simulations, the anisotropic dielectric properties of CLCs were incorporated through their permittivity tensor ε, expressed as:

(S1)

where $\varepsilon_{x}$​, $\varepsilon_{y}$, and $\varepsilon_{z}$represent the principal dielectric constants along the x-, y-, and z-axes, respectively.

To account for the spatial orientation of the LCs molecules, the permittivity tensor ε was rotated according to the director field, which represents the local molecular orientation of the LCs. The transformed permittivity tensor ε' is given by:

(S2)

where $\mathbf{R}$is the rotation matrix determined by the orientation of the LCs director.

Considering the spatial scale and symmetry of the structure, 2D simulations were employed to significantly reduce computational costs while ensuring simulation accuracy. In our simulations, the LCs director undergoes periodic spatial rotation around the y-axis. The corresponding rotation matrix is expressed as:

$\mathbf{R}=\left[\begin{matrix} \cos\theta& 0 & \sin\theta\\ 0 & 1 & 0 \\ -\sin\theta& 0 & \cos\theta\end{matrix}\right]$ (S3)

where the rotation angle $\theta$ varies as a function of the y-coordinate:

$\theta=\frac{2\pi y}{\text{pitch}}$ (S4)

Here, pitch denotes the periodicity of the LCs director field along the y-axis.

**Figures S20b** and **S20c** present the normalized far-field transmittance results for CLCs with pitches of 332.5 nm and 375 nm, respectively. The simulation results show excellent agreement with the experimental measurements.

To simulate the dipole orientations of various QEs, dipole sources were employed, as illustrated in **Figure S20a**. To account for the incoherent nature of the light source, independent simulations were conducted for dipoles oriented along different horizontal directions. The final field intensity was determined by averaging the results of these simulations. The angular distribution of radiance, *Rad (θ, λ)*, which captures the contributions of different proportions of in-plane (IP) and out-of-plane (OP) dipoles, was simulated using a weighted combination of IP and OP dipoles. In our simulations, QDs with random dipole orientations were modeled using 67% IP dipoles and 33% OP dipoles. Self-assembled face-down oriented CQWs were modeled using 100% IP dipoles.

To compute the left circularly polarized luminescence (L-CPL) and right circularly polarized luminescence (R-CPL) emitted from the structure, we first extracted the s- and p-polarized electric fields ($E_{s}$and $E_{p}$*​*​) in quartz (after passing through the CLCs) from the FDTD solutions, as shown in **Figure S21**. The electric field amplitudes for L-CPL and R-CPL were calculated using the following equations:

$E_{\text{L-CPL}}=\frac{1}{\sqrt{2}}\left(E_{s}+iE_{p}\right), E_{\text{R-CPL}}=\frac{1}{\sqrt{2}}\left(E_{s}-iE_{p}\right)$ (S5)

where $E_{L-CPL}$and $E_{R-CPL}$ ​represent the field amplitudes of L-CPL and R-CPL, respectively.

The intensities of L-CPL and R-CPL within the quartz substrate are then calculated as:

$I_{\text{L-CPL}}=|E_{\text{L-CPL}}|^{2}, I_{\text{R-CPL}}=|E_{\text{R-CPL}}|^{2}$ (S6)

The *Rad (θ, λ)* of L-CPL and R-CPL in quartz is normalized as:

(S7)

where:

- $\varepsilon_{0}$​ and $\mu_{0}$​ are the vacuum permittivity and permeability, respectively.
- $n_{1}$is the refractive index of quartz.
- sourcepower(λ) is the source power at wavelength λ.

The $g_{lum}$ was then calculated using the following equation:

$g_{\text{lum}}=\left|\frac{2\cdot\left(Rad_{\text{L-CPL, Quartz}}-Rad_{\text{R-CPL, Quartz}}\right)}{Rad_{\text{L-CPL, Quartz}}+Rad_{\text{R-CPL, Quartz}}}\right|$ (S8)


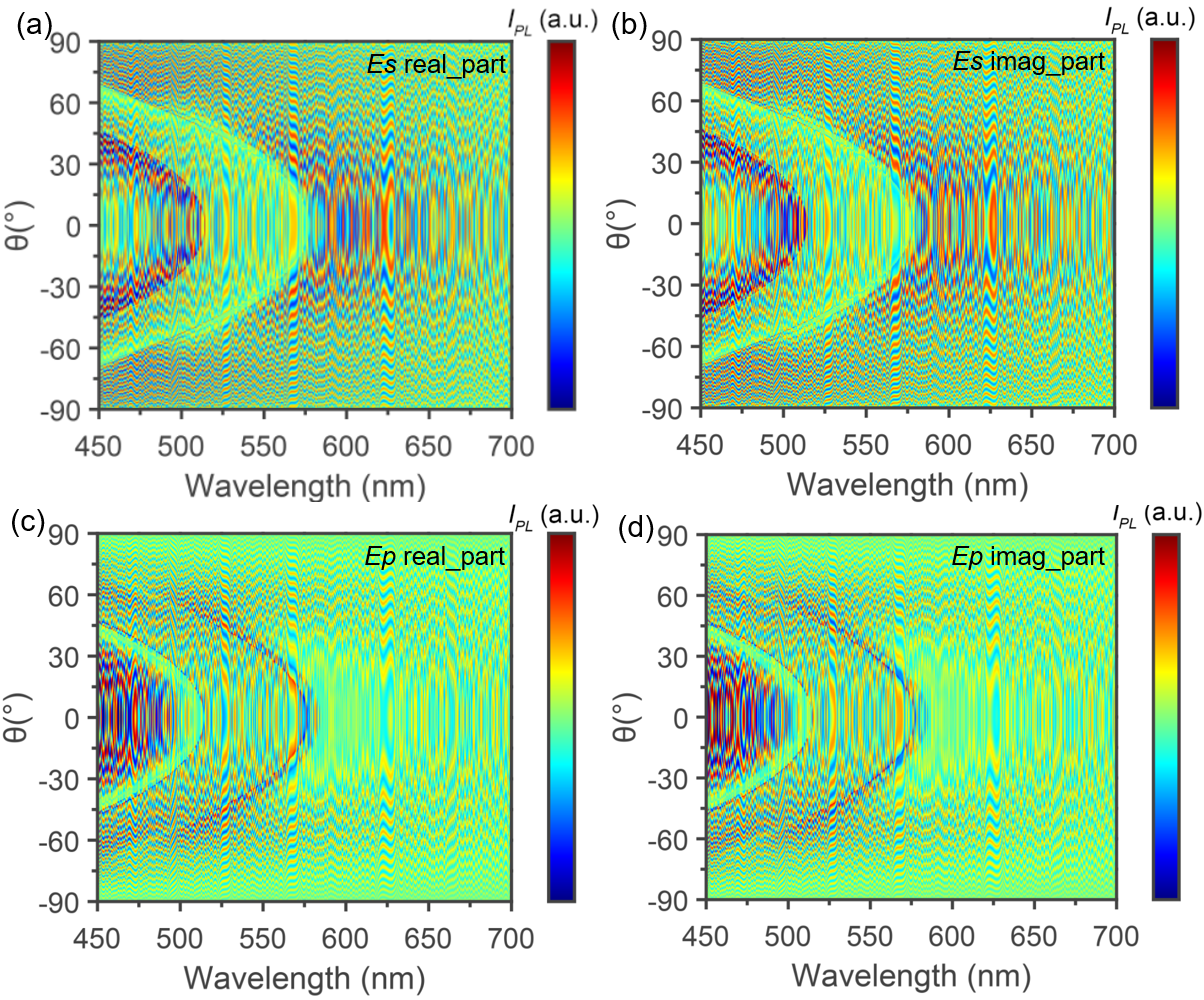


**Figure S21.** The simulated (a, c) real parts and (b, d) imaginary parts of $E_{s}$ and $E_{p}$ from the far field results in quartz, respectively.

The emission spectrum of QEs with different linewidths was modeled using a Gaussian function:

$I(\lambda)=I_{0}\exp\left(-\frac{(\lambda-\lambda_{0})^{2}}{2\sigma^{2}}\right)$ (S9)

where$I(\lambda)$is the intensity at wavelength$\lambda$, $I_{0}$*​* is the peak intensity, $\lambda_{2}$is the peak wavelength, and $\sigma$is the standard deviation related to FWHM.

To reasonably compare the intensity of circularly polarized light ultimately converted from different emissive materials, we control the initial intensity of the materials to be the same. The spectral emission intensity curve of an emissive material typically represents the radiative power (or photon flux) per unit wavelength range. The total radiative power or total emitted energy over the entire spectrum can be calculated by integrating the spectral curve $I(\lambda)$ over the wavelength range [$\lambda_{1}$,$\lambda_{2}$​]:

$P_{QE}=\int_{\lambda_{1}}^{\lambda_{2}} I(\lambda) d\lambda$ (S10)

By imposing the condition that the integrated area of the spectral curve is the same, we ensure that emissive materials with different spectral shapes have the same total emission intensity, and the corresponding *I(θ)* of L-CPL *and* R-CPL in the top glass substrate were given by:

$I_{L-CPL,Quartz}(\theta)=\int_{\lambda_{1}}^{\lambda_{2}} Rad_{L-CPL,Quartz}\cdot I(\lambda), I_{R-CPL,Quartz}(\theta)=\int_{\lambda_{1}}^{\lambda_{2}} Rad_{R-CPL,Quartz}\cdot I(\lambda)$ (S12)

The angular-dependent $g_{lum}$​ within this medium was calculated as:

$g_{\text{lum}}(\theta)=\left|\frac{2\cdot\left(I_{\text{L-CPL, Quartz}}-I_{\text{R-CPL, Quartz}}\right)}{I_{\text{L-CPL, Quartz}}+I_{\text{R-CPL, Quartz}}}\right|$ (S13)

The intensities of L-CPL and R-CPL transmitted from quartz into air were calculated using Fresnel coefficients and Snell’s law. The Fresnel transmission coefficients for s- and p-polarized light ($T_{s}$and$T_{p}$) were applied as follows:

$I_{\text{L-CPL, air}}(\theta)=\frac{n_{1}}{n_{\text{air}}}\cdot0.5\cdot(T_{s}+T_{p})\cdot I_{\text{L-CPL, Quartz}}\cdot\frac{d\theta_{1}}{d\theta_{2}},\text{ }I_{\text{R-CPL, air}}(\theta)=\frac{n_{1}}{n_{\text{air}}}\cdot0.5\cdot(T_{s}+T_{p})\cdot I_{\text{R-CPL, Quartz}}\cdot\frac{d\theta_{1}}{d\theta_{2}}$ (S14)

The angular-dependent $g_{lum}$​ in free space was then calculated using the following equation:

$g_{\text{lum}}(\theta)=\left|\frac{2\cdot\left(I_{\text{L-CPL, air}}-I_{\text{R-CPL, air}}\right)}{I_{\text{L-CPL, air}}+I_{\text{R-CPL, air}}}\right|$ (S15)


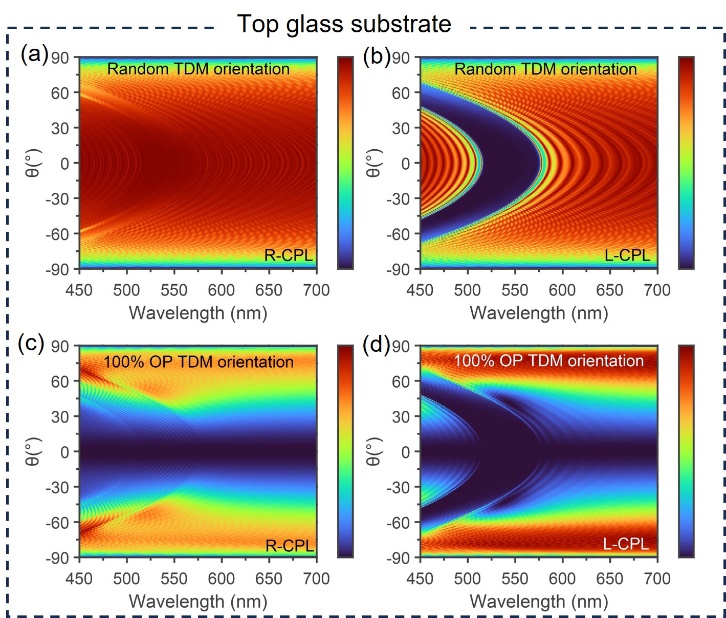


**Figure S22.** Supplementary radiance pattern within in the medium of top glass substrate. Simulated radiance pattern for (a) R-CPL and (b) L-CPL from QEs with random TDM orientation. Simulated radiance pattern for (a) R-CPL and (b) L-CPL from QEs with 100% OP TDM orientation.


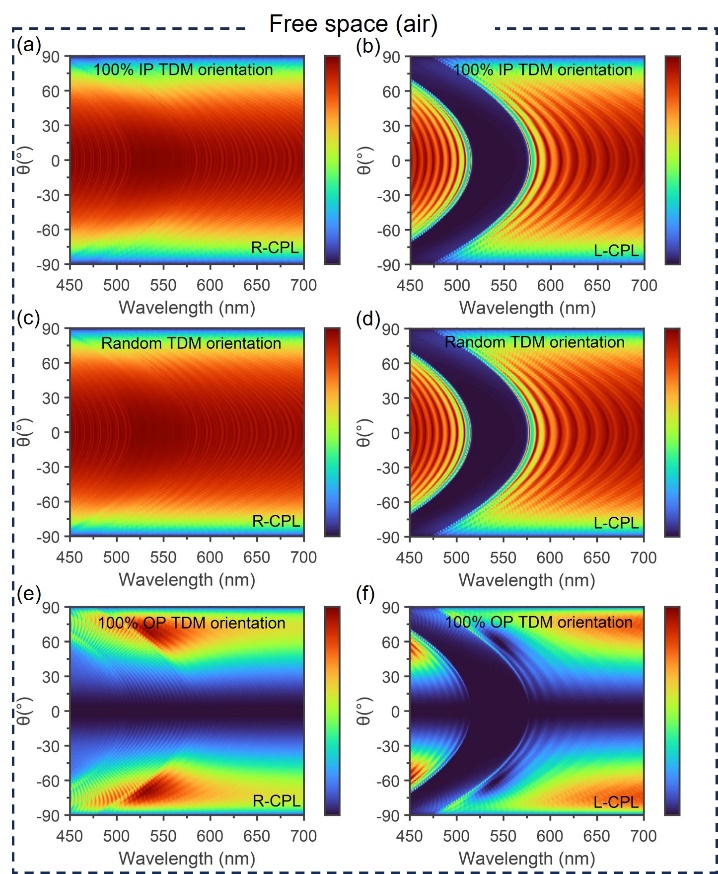


**Figure S23**. Supplementary radiance pattern in free space. Simulated radiance pattern for (a) R-CPL and (b) L-CPL from QEs with 100% IP TDM orientation. Simulated radiance pattern for (c) R-CPL and (d) L-CPL from QEs with random TDM orientation. Simulated radiance pattern for (e) R-CPL and (f) L-CPL from QEs with 100% OP TDM orientation.

**Section 7. Additional information for demonstrations.**


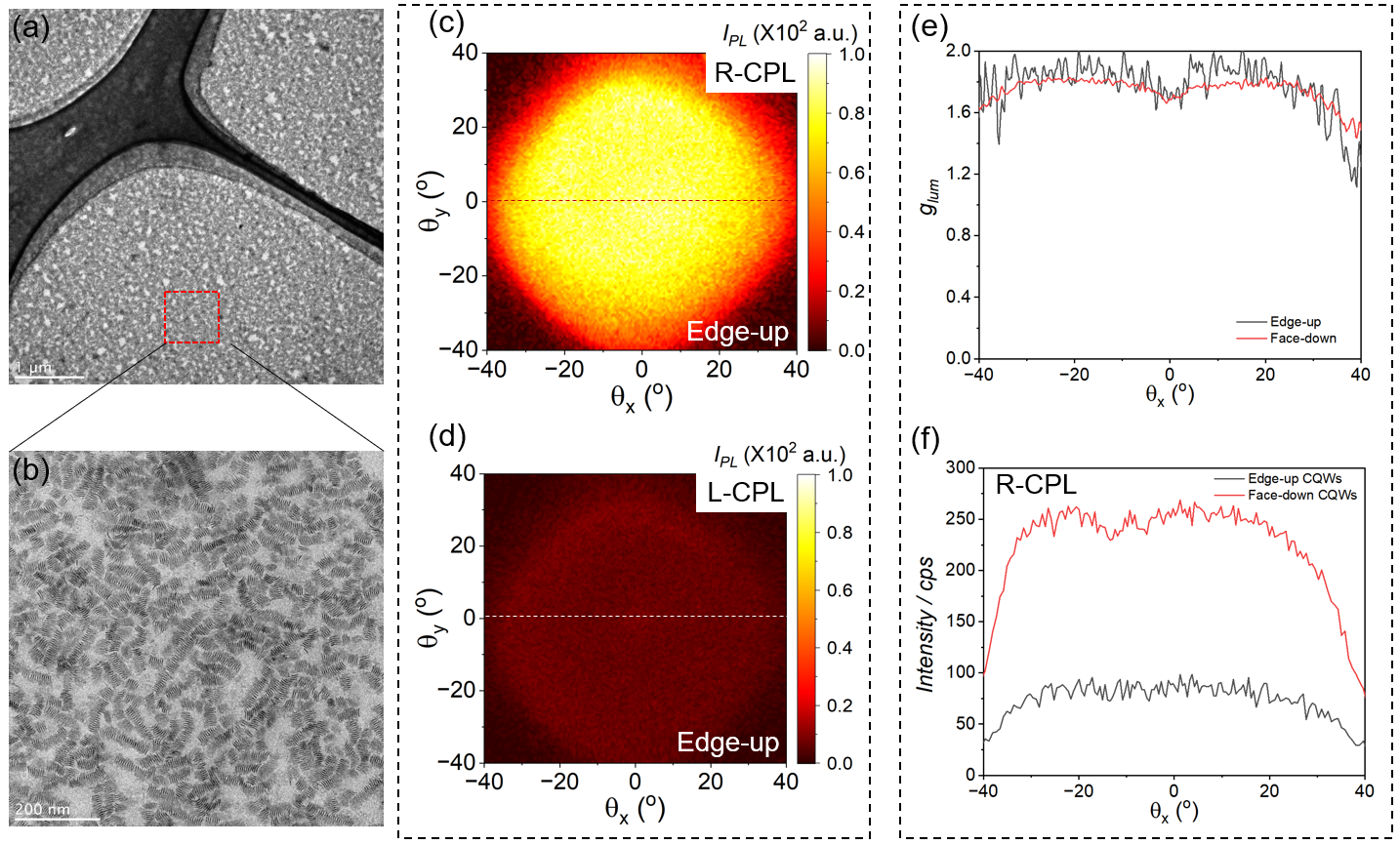


**Figure S24.** (a, b) TEM images showing the edge-up self-assembly of CQWs. (c, d) BFP images of the edge-up self-assembled CQWs/CLCs sample, filtered through (c) right-handed and (d) left-handed circular polarizers, respectively. (e) Comparison of the angular distribution of *g_lum_* between face-down (red line) and edge-up (black line) self-assembled CQWs/CLCs samples. (f) Comparison of right-handed CPL emission intensity between face-down (red line) and edge-up (black line) self-assembled CQWs/CLCs samples.


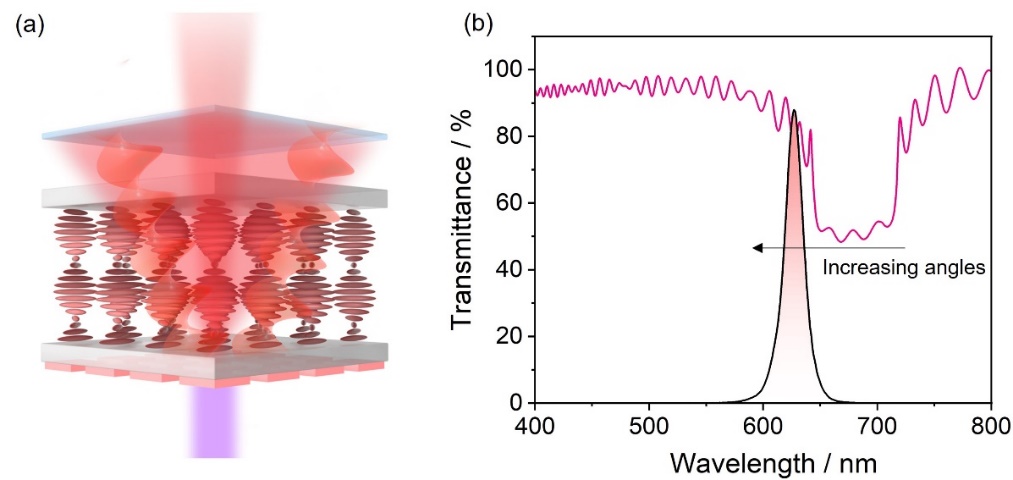


**Figure S25.** (a) Schematic representation and (b) relationship between the reflection band of the CLCs and the PL spectra of red-emitting CQWs.


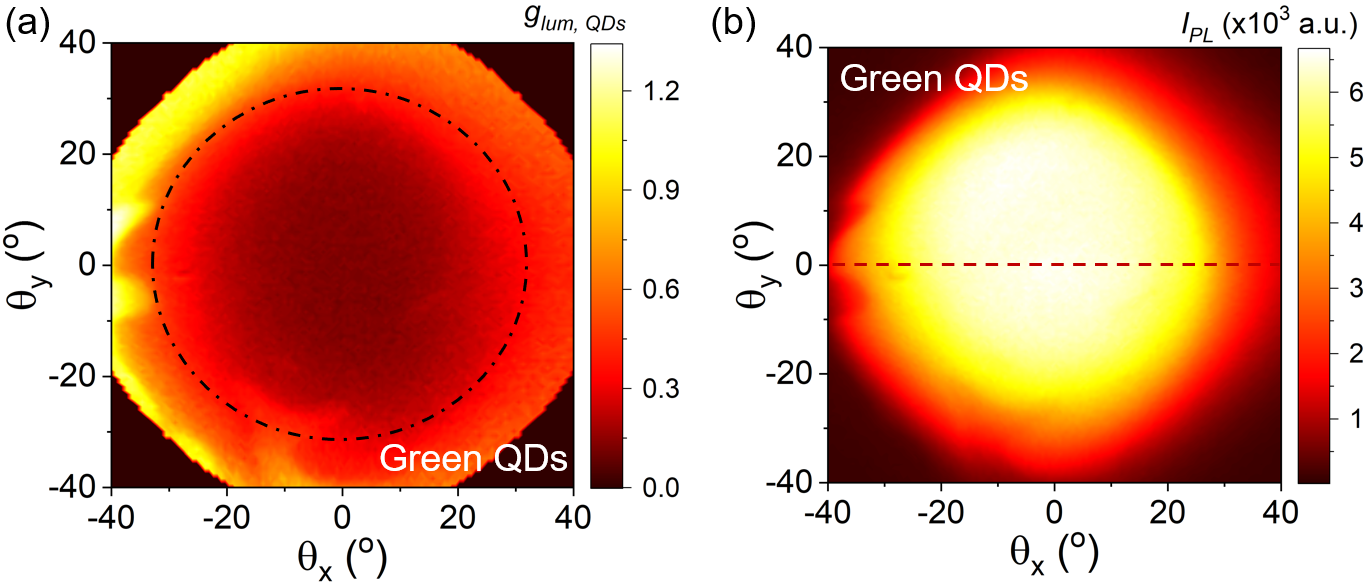


**Figure S26.** (a) The calculated $g_{lum}$​ and the (b) corresponding BFP image of the anti-peep sample from QDs/CLCs.


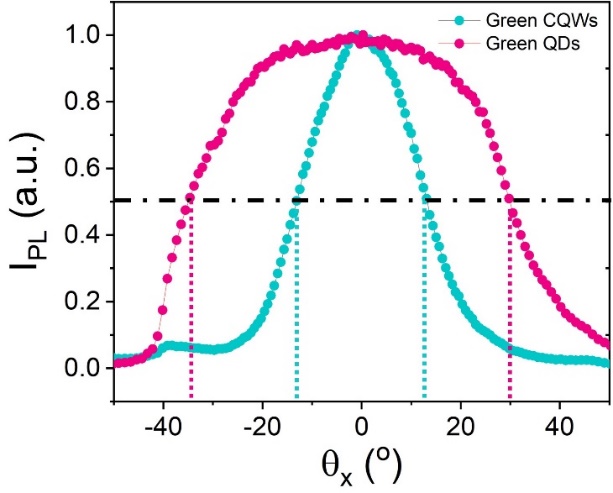


**Figure S27.** Normalized cross-sectional intensity profiles along θ_x_ for the green CQWs/CLCs and green QDs/CLCs samples.

**
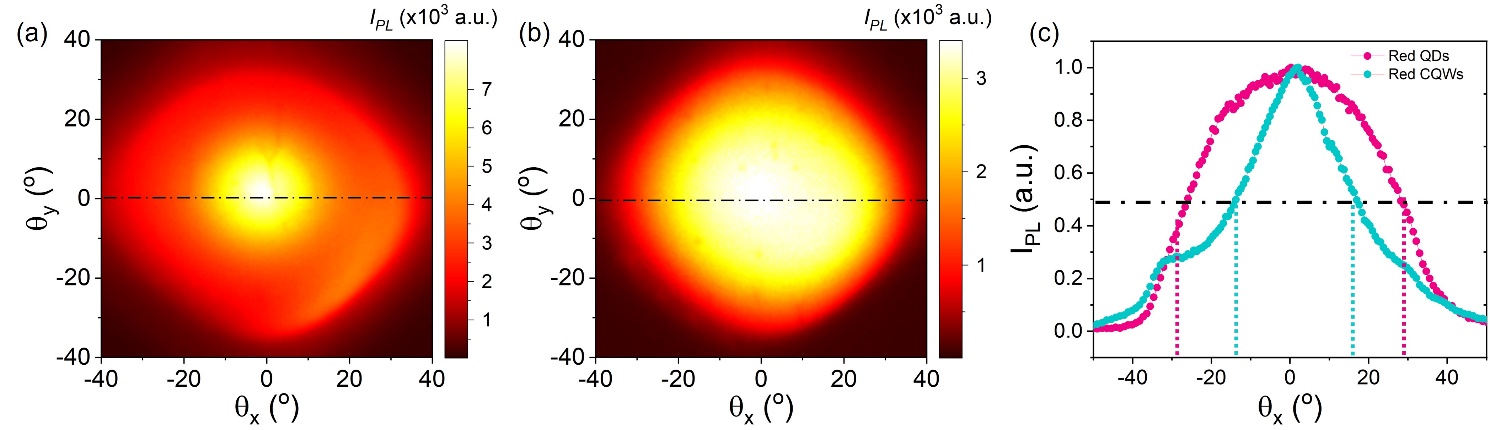
**

**Figure S28.** BFP images of the anti-peep samples from (a) red CQWs/CLCs and (b) red QDs/CLCs. (c) Normalized cross-sectional intensity profiles along θ_x_ for the red CQWs/CLCs and red QDs/CLCs samples


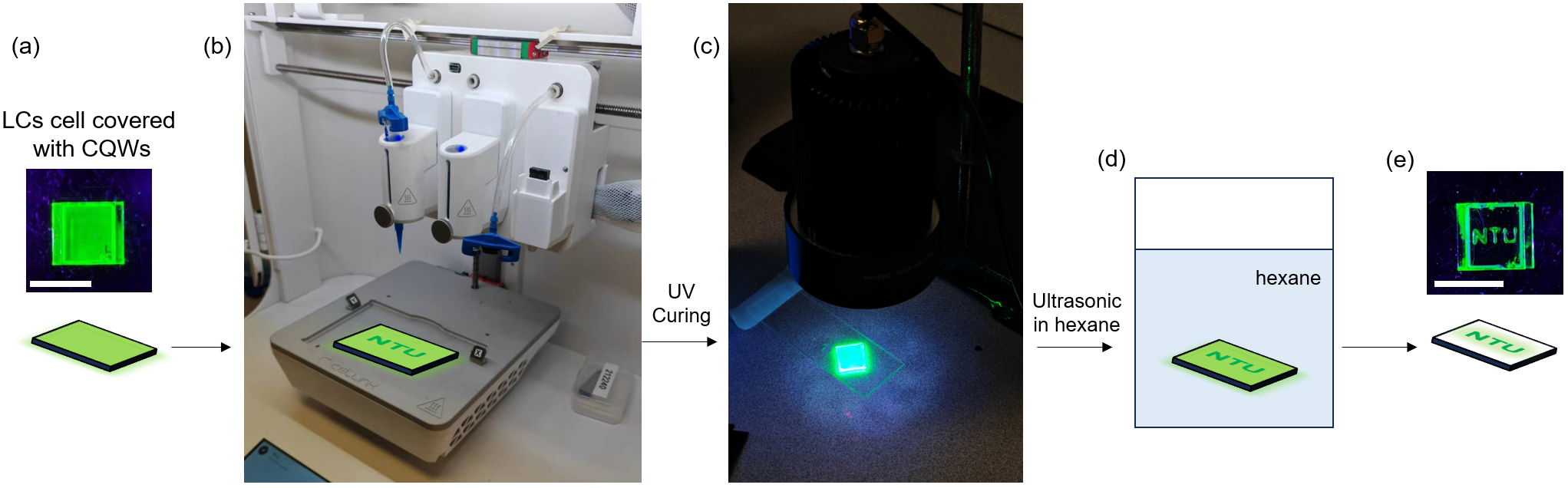


**Figure S29.** Schematic of the fluorescent pattern fabrication process facilitated by 3D printing: (a) CQWs are self-assembled and deposited on the surface of a CLCs cell. Scale bar, 1 cm. (b) a polymer precursor is printed onto the CQWs/CLCs in predefined patterns. (c) UV irradiation induces polymerization, anchoring the CQWs to the patterned polymer. (d,e) The sample is then immersed in hexane and sonicated to remove uncovered CQWs, resulting in a fluorescent pattern. Scale bar, 1 cm.

**Reference**

[1] Y. Altintas, U. Quliyeva, K. Gungor, O. Erdem, Y. Kelestemur, E. Mutlugun, M. V. Kovalenko, H. V. Demir, *Small* **2019**, 15, 1804854.

[2] a) P. Bai, A. Hu, Y. Deng, Z. Tang, W. Yu, Y. Hao, S. Yang, Y. Zhu, L. Xiao, Y. Jin, *The Journal of Physical Chemistry Letters* **2022**, 13, 9051; b) A. Hu, P. Bai, Y. Zhu, Z. Tang, L. Xiao, Y. Gao, *Small* **2022**, 18, 2204120.

[3] G. H. Bertrand, A. Polovitsyn, S. Christodoulou, A. H. Khan, I. Moreels, *Chemical Communications* **2016**, 52, 11975.

[4] W. K. Bae, J. Kwak, J. W. Park, K. Char, C. Lee, S. Lee, *Advanced Materials* **2009**, 21, 1690.

[5] X. Liang, S. Guo, M. Chen, C. Li, Q. Wang, C. Zou, C. Zhang, L. Zhang, S. Guo, H. Yang, *Materials Horizons* **2017**, 4, 878.

[6] J. D. Lin, T. Y. Wang, T. S. Mo, S. Y. Huang, C. R. Lee, *Scientific Report* **2016**, 6, 30407.
